# Climate change impact on flood and extreme precipitation increases with water availability

**DOI:** 10.1038/s41598-020-70816-2

**Published:** 2020-08-13

**Authors:** Hossein Tabari

**Affiliations:** Department of Civil Engineering, KU Leuven, Leuven, Belgium

**Keywords:** Climate change, Natural hazards

## Abstract

The hydrological cycle is expected to intensify with global warming, which likely increases the intensity of extreme precipitation events and the risk of flooding. The changes, however, often differ from the theorized expectation of increases in water‐holding capacity of the atmosphere in the warmer conditions, especially when water availability is limited. Here, the relationships of changes in extreme precipitation and flood intensities for the end of the twenty-first century with spatial and seasonal water availability are quantified. Results show an intensification of extreme precipitation and flood events over all climate regions which increases as water availability increases from wet to dry regions. Similarly, there is an increase in the intensification of extreme precipitation and flood with the seasonal cycle of water availability. The connection between extreme precipitation and flood intensity changes and spatial and seasonal water availability becomes stronger as events become less extreme.

Extreme precipitation is expected to intensify with global warming over large parts of the globe as the concentration of atmospheric water vapour which supplies the water for precipitation increases in proportion to the saturation concentrations at a rate of about 6–7% per degree rise in temperature according to the thermodynamic Clausius–Clapeyron relationship^1–3^. However, changes in atmospheric dynamics such as poleward expansion of tropical Hadley circulation can weaken^4–6^ or reinforce^7^ the thermodynamic effect regionally and modify the extreme precipitation amplification. Water availability also plays a large role in the moisture–temperature relationship^8^.

Due to different interacting drivers of extreme precipitation changes, the changes are not uniform in space and vary by region^9^. The scaling rate of extreme precipitation with land surface temperature is not accordingly constant. Even a negative scaling at higher temperatures has been observed in some places^10^, which has been suggested to be a result of limited moisture availability^11^ or arid surface conditions^12^. Whereas in wet regions amplified atmospheric moisture convergence can intensify the effects of extreme precipitation, in dry environments precipitation increases may be counteracted by evaporation^13^. Recent studies have examined daily extreme precipitation changes in relation to water availability and found that 30-year averaged annual precipitation maxima aggregated over the dry and wet regions of the world is likely to increase^14,15^. As rarer precipitation events are expected to be more influenced by climate change^7,16,17^ and scale with vertical moisture transport rather than horizontal moisture advection^6^, it remains unresolved whether the relationships between extreme precipitation changes and water availability can also be detectable for rare flood-producing precipitation events.

Using gridded observations in Europe, a positive scaling rate of extreme precipitation with temperature in winter and a negative one in summer has been reported^18^. This raises the question of whether extreme precipitation changes have any relation with the seasonal cycle of water availability in a similar fashion as regional water availability. Understanding of the relationships between the climate change impact on extreme events and water availability is essential in the future-proofed planning for global change in different climate regimes to ensure a sustainable socioeconomic development at the regional scales.

Extreme precipitation amplification may increase the intensity and frequency of flooding, imposing heavy costs to aquatic and terrestrial ecosystems, human societies and the economy. Changes in flood characteristics not only depend on the spatial distribution, time evolution and rarity of precipitation^19^, but on antecedent soil moisture conditions and in snow-dominated regions on snowmelt timing^20^ and snowpack magnitude^21^. Global-scale flood assessments have reported both decreases and increases in future floods under global warming^22–25^, albeit by using varying hydrological and climate models, scenarios, bias-correction methods and flood indicators which hinders drawing a common perspective of future flood changes^26^. Changes in soil moisture and runoff have been shown to correlate well with changes in climatic moisture at the regional scale^27,28^. More significant changes have also been found in observed annual maximum flows in wet regions than in dry regions^29^. This is because in the former a higher fraction of precipitation changes leads to runoff changes, while in the latter a large buffer is available to dampen precipitation changes causing smaller runoff changes^30–32^. Owing to the complex mechanisms of flood changes, it is not known whether relationships between extreme precipitation changes and water availability can be generalized for flood changes.

Here, the relationships of future changes in extreme precipitation and flood intensities with water availability are analyzed. Extreme precipitation changes per K global warming in 2070–2099 under the RCP8.5 scenario compared with 1971–2000 are computed using simulations of 24 global climate models (GCMs) from the Coupled Model Intercomparison Project Phase 5 (CMIP5^33^) and flood changes using simulations from multi-model ensemble of five global impact models (IMs) and four CMIP5 GCMs (20 IM-GCM combinations) from the Inter-Sectoral Impact Model Intercomparison Project (ISIMIP^34^). The climatological water availability is determined based on the aridity index (AI), as the ratio between potential evapotranspiration (water demand) and precipitation (water supply), for historical and future simulations of individual CMIP5 models (see “Methods” for details). It ensures that the expected changes in the geographical location of the climatic regions with global warming and the discrepancy among models are taken into account.

## Results and discussion

### Relationships of flood and extreme precipitation changes with spatial water availability

**Figure 1:**
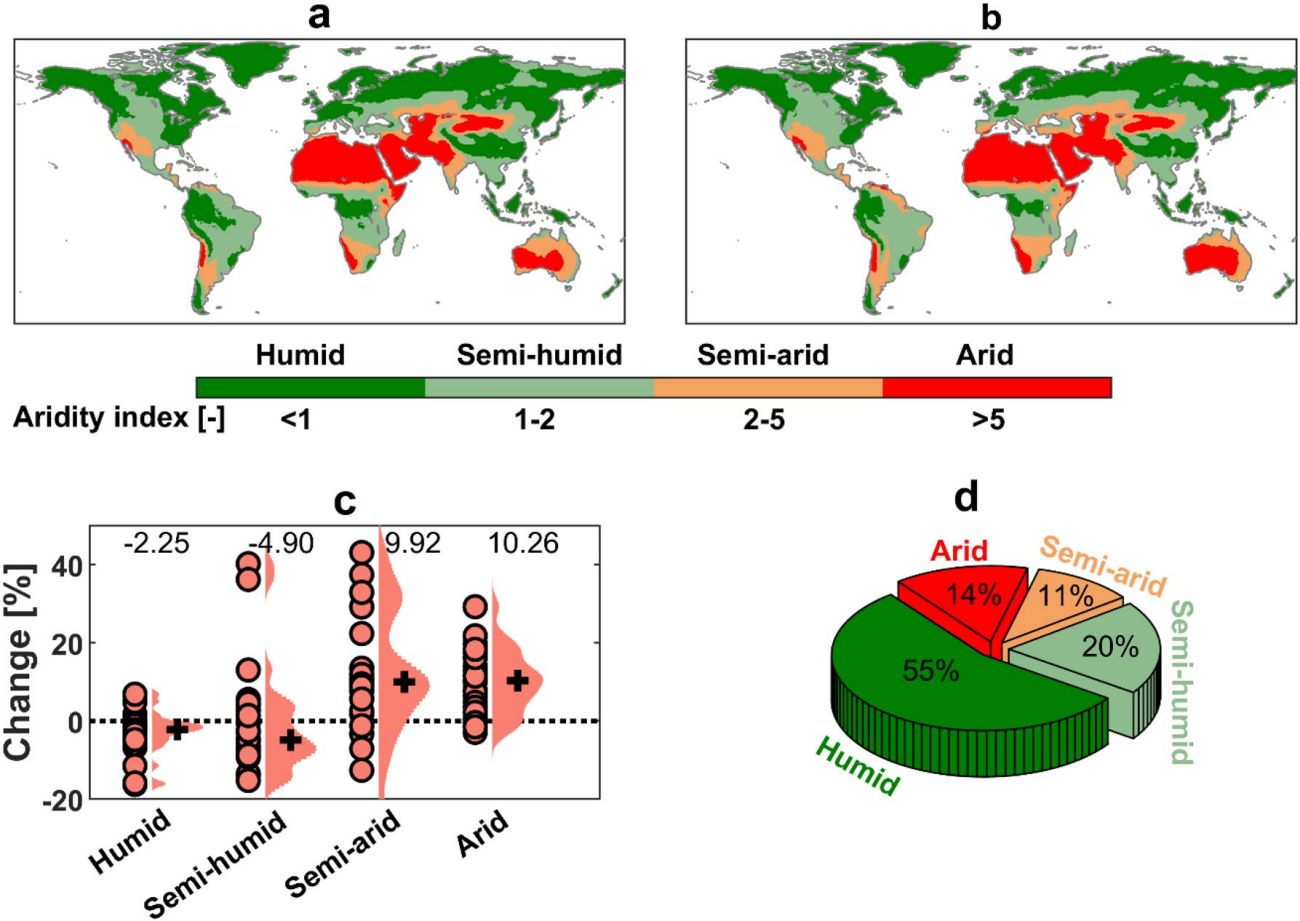
Aridity index and its expected future changes. (**a**,**b**) Spatial distribution of ensemble median aridity index and five climate regimes based on the aridity index of the (**a**) historical (1971–2000) and (**b**) future (2070–2099) climates. (**c**) Projected change in area coverage of each climate regime for the period 2070–2099 with respect to the reference 1971–2000 (salmon dots, individual models); ensemble median is shown by black cross and number at the top. (**d**) Ensemble median projected area coverage of each of the five climate regimes in percent of total terrestrial land area for 2070–2099. The maps were generated using the MATLAB mapping toolbox^65^ (URL https://www.mathworks.com/products/mapping.html).

Based on the ensemble median of the CMIP5 GCMs, water-limited regions are mainly located in North Africa & the Middle East (MENA) and Australia, while water-abundant regions are located in the mid-latitudes and the tropics (Fig. 1a). About 72% of the global land is likely to undergo aridification in the future, with substantial aridification (aridity increase of > 30%) in MENA, south Europe, south Africa and Australia (Fig. S3), leading to a shift in climate regimes (Fig. 1b). Globally, arid and semi-arid regions would expand by 10.3% and 9.9%, respectively, while humid and semi-humid regions would decrease by 2.3% and 4.9%, respectively (Fig. 1c). It makes the area coverage of humid, semi-humid, semi-arid and arid climates at the end of the twenty-first century equal to 55%, 20%, 11% and 14% of the total terrestrial land area, respectively (Fig. 1d).

There is a consistency between most CMIP5 GCMs on the geographical distribution of climate regimes (Figs. S4 and S5). IPSL models are the ones with the largest difference from the other models, which simulate much larger water-limited regions. Because of this discrepancy among GCMs on the location of different climate regimes as well as the expected shift in the regimes in the future, the relationships of extreme precipitation and flood changes with water availability are investigated based on model-specific masks of dynamic (future) climate regimes.

**Figure 2:**
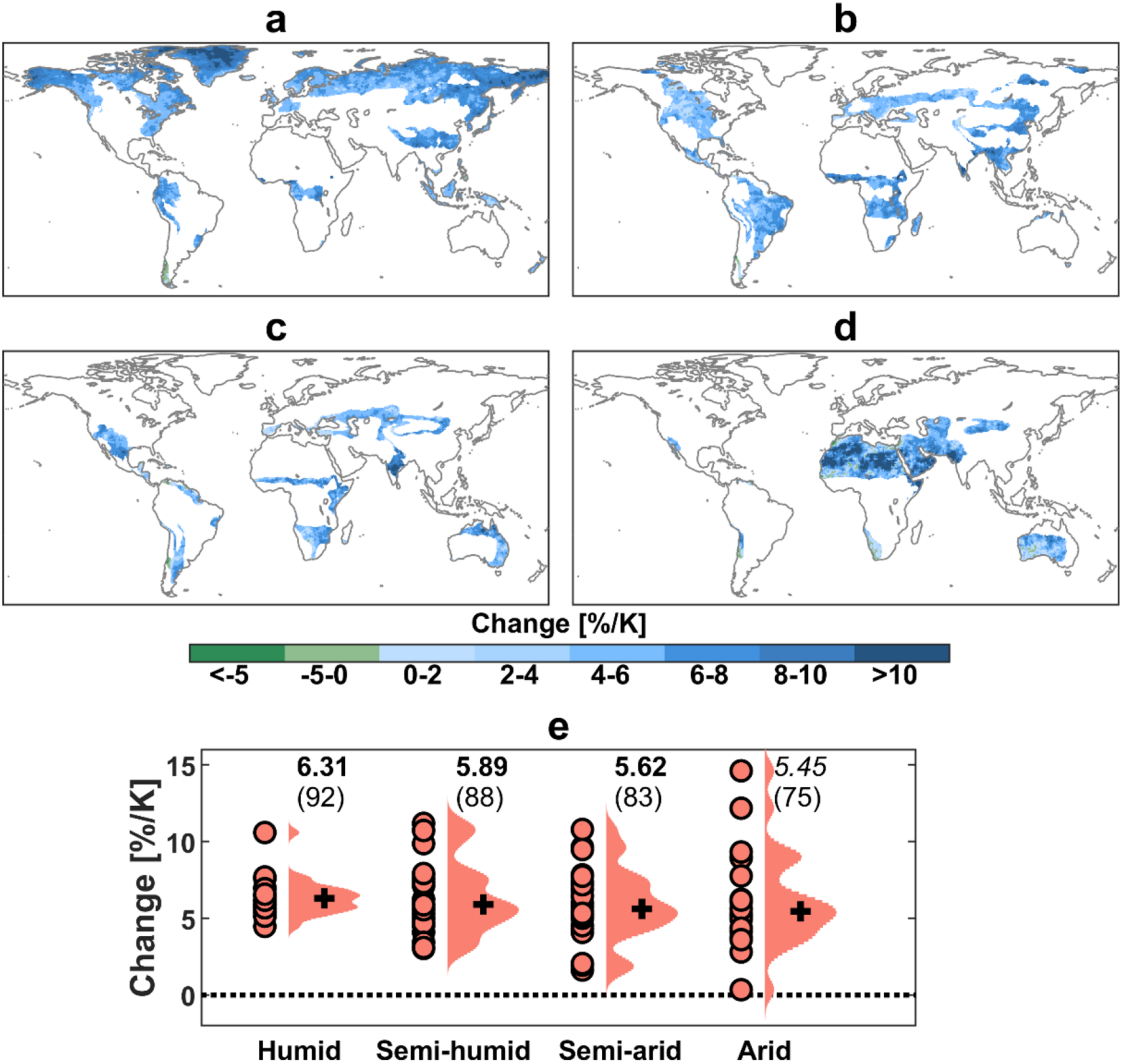
Changes (%) in 1-in-30-year extreme precipitation intensity per K global warming in 2070–2099 under RCP8.5, compared with 1971–2000. (**a**–**d**) Spatial distribution of ensemble median changes in (**a**) humid, (**b**) semi-humid, (**c**) semi-arid and (**d**) arid regions. (**e**) Changes per climate regime based on individual models (salmon dots). For each violin, ensemble median is shown by black cross. The numbers on top of the violins (top row) indicate ensemble median and those in bold face and italic denote significant changes at the 95% and 90% confidence levels, respectively. The numbers in brackets indicate the percentage of experiments that agree on the sign of change (robustness). The maps were generated using the MATLAB mapping toolbox^65^ (URL https://www.mathworks.com/products/mapping.html).

The spatial distribution of changes in 1-in-30-year extreme precipitation intensity shows that extreme precipitation increases uniformly across all the climate regimes (Fig. 2), in agreement with previous findings for rare extreme events^35,36^. Precisely, 99.9%, 99.8%, 99.3% and 98.7% of land area respectively in humid, semi-humid, semi-arid and arid regions show an increase in extreme precipitation intensity (Fig. 2). There is, nevertheless, a substantial uncertainty in the multi-model median changes in Latin America, Africa, the Middle East and Australia (Figs. S5 and S7a). This is in line with the uncertainty hotspots of less extreme precipitation identified in earlier studies^9^. A large uncertainty in the uncertainty hotspots has been attributed to the convective nature of rainstorms which cannot be adequately represented and resolved by coarse-scale GCMs, and to a sparse observational network which hinders the tuning and improvement of GCMs over these regions^9^.

As the particular focus of this study is to investigate extreme precipitation and flood changes with water availability, changes are analyzed with respect to global climate regimes. Extreme precipitation in humid regions increases with global warming (6.31%/K) at the Clausius–Clapeyron rate (Fig. 2e). The extreme precipitation increase in humid regions is significant at the 5% level and robust where the increasing signal is projected by 92% of the GCMs. For semi-humid regions, the increase is smaller at the rate of 5.89%/K which is significant at the 5% level and robust (88% of the GCMs). Moving towards more water-limited regions, the extreme precipitation-temperature scaling rate further decreases; 5.62 and 5.45%/K for semi-arid and arid regions, respectively. The extreme precipitation increase is significant and robust for both semi-arid and arid regions, although with a lower level of robustness and significance. In the mid-to-high latitudes which occupy a large portion of global humid and semi-humid regions, extreme precipitation changes are mainly controlled by the thermodynamic effect^4–6^. In low latitudes where most arid regions are located, the intensifying tendency of extreme precipitation originating from thermodynamics is offset by the dynamic effect^4–6^.

The total uncertainty in extreme precipitation changes indeed depends on climatic regime and increases with decreasing water availability (Fig. S8a,c). Earlier studies have also found a larger spread of extreme precipitation changes in dry regions compared wet regions^14,37^. The increasing total uncertainty with decreasing water availability is attributed to the increasing trend of both its components (GCM and hazard quantification method); however, the GCM uncertainty has a much larger contribution in all regions.

**Figure 3:**
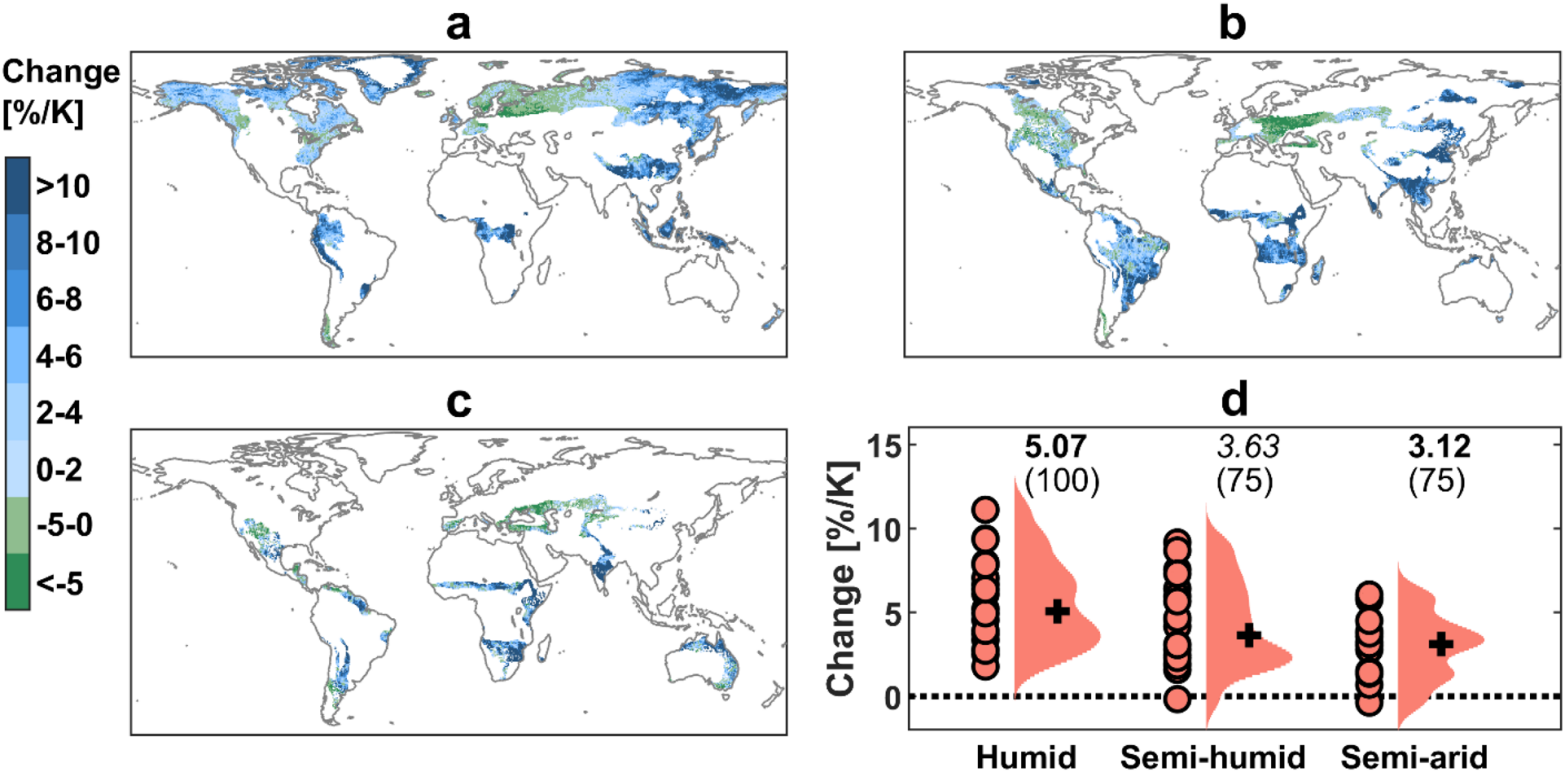
Changes (%) in 1-in-30-year flood intensity per K global warming in 2070–2099 under RCP8.5, compared with 1971–2000. (**a**–**c**) Spatial distribution of ensemble median changes in (**a**) humid, (**b**) semi-humid and (**c**) semi-arid regions. (**d**) Changes per climate regime based on individual experiments (salmon dots). For each violin, ensemble median is shown by black cross. The numbers on top of the violins (top row) indicate ensemble median and those in bold face and italic denote significant changes at the 95% and 90% confidence levels, respectively. The numbers in brackets indicate the percentage of experiments that agree on the sign of change (robustness). Grid cells with annual maxima close to 0 m^3^ s^−1^ of the historical model period are screened out. The maps were generated using the MATLAB mapping toolbox^65^ (URL https://www.mathworks.com/products/mapping.html).

Flood intensity is projected to increase over most areas of the globe (Fig. 3), with a large uncertainty in some places (Figs. S7b and S10). 75.9% of land area in humid regions shows an increase in flood intensity, while semi-humid and semi-arid regions show a lower percentage of land area with increasing flood intensity, accounting for 68.7% and 63.4%, respectively (Fig. 3). Flood changes follow the extreme precipitation change direction over regions where precipitation plays the dominant role in flood occurrence; however, inconsistent changes are found where there are other flood generating factors in play (Fig. S10). Decrease in flood intensity is observed in snow dominated regions (e.g., North and Central Europe) where spring snowmelt is decreasing under global warming^38^ or in regions (e.g., Mediterranean) where annual precipitation is projected to decrease^39,40^ and where antecedence soil moisture plays a significant role in flood generations^19,41^.

Aggregated over different climate regimes, broadly similar pattern to extreme precipitation changes is obtained for flood changes where flood intensity increases in all climate regimes with the magnitude increasing with water availability (Fig. 3d). As most of arid regions are masked out for the flood analysis, the relationship of flood changes with water availability is examined by comparing the results among humid, semi-humid and semi-arid climate regimes. Flood intensity increases at the rates of 5.07, 3.63 and 3.12%/K for humid, semi-humid and semi-arid climate regions, respectively. The increase for all regions is significant and robust with an agreement among > 75% of the experiments. The uncertainty in projected flood intensity changes increases as the climate gets drier (Fig. S8b,d). It highlights the necessity of using large multi-model ensembles including multiple impact models forced by several climate models for hydrological climate change analyses in drier regions. For all climate regimes, GCMs are the main contributor to the flood change uncertainty, while hazard quantification methods have the lowest contribution. IM uncertainty in humid regions is larger than global average IM uncertainty. The dominance of the GCM uncertainty corroborates the findings of Hagemann et al.^42^ and Giuntoli et al.^43^ who showed a larger contribution of climate model uncertainty compared to global hydrological models.

The results also show that using the median ensemble mask of climate regimes instead of model-specific masks and assuming static climate regimes instead of dynamic ones would lead to bias in extreme precipitation and flood intensity changes for different climate regions (see Texts S3 and S4 and Figs. S11 and S12 for more details). The bias increases towards drier climates and is larger in the case of using the median ensemble mask of climate regimes compared to static climate regimes.

### Relationships of flood and extreme precipitation changes with the seasonal cycle of water availability

**Figure 4:**
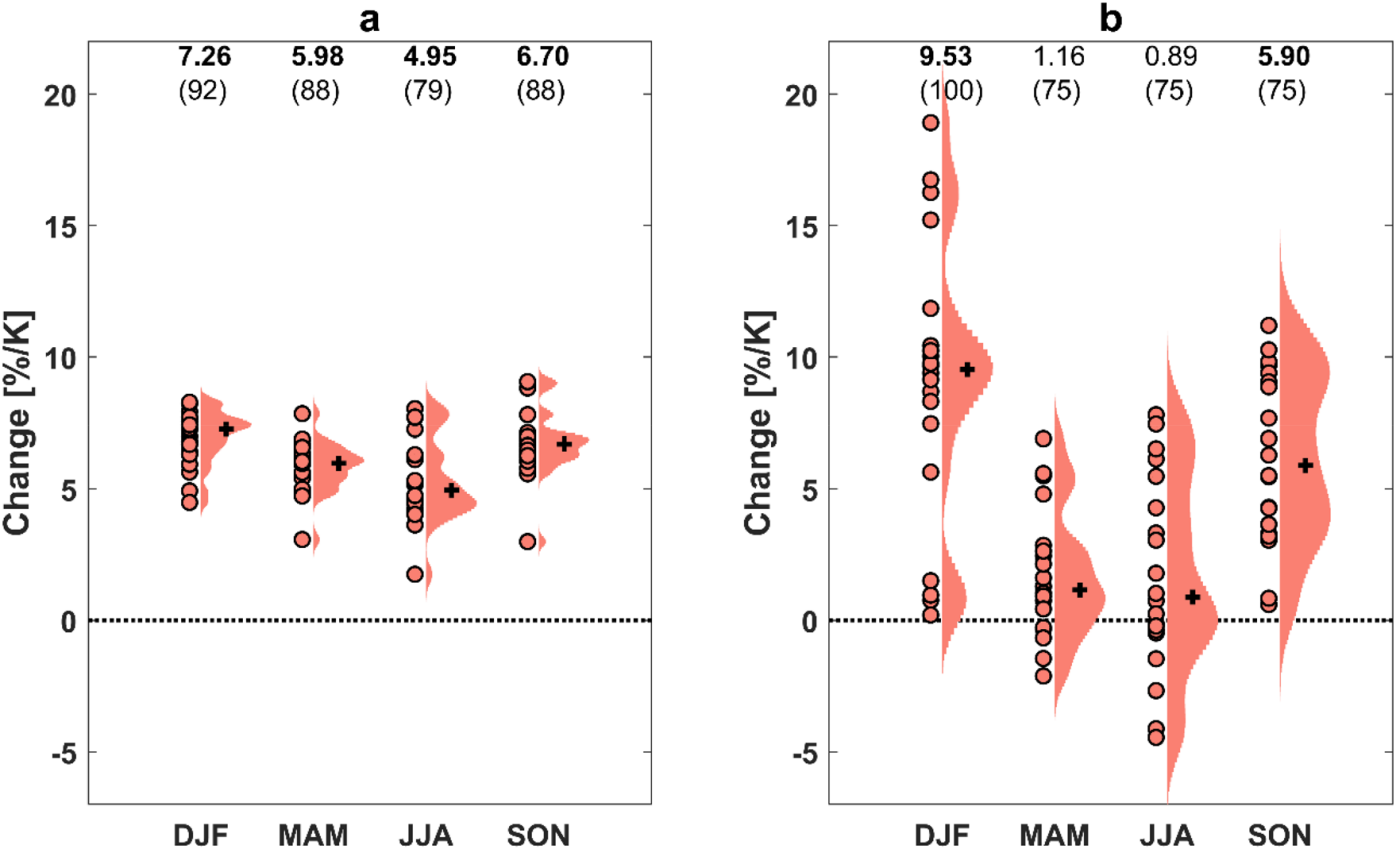
Changes in 1-in-30-year (**a**) extreme precipitation and (**b**) flood intensity per season (salmon dots, individual models) in the Northern Hemisphere mid-latitudes. For each violin, ensemble median is shown by black cross. The numbers on top of the violins (top row) indicate ensemble median and those in bold face and italic denote significant changes at the 95% and 90% confidence levels, respectively. The numbers in brackets indicate the percentage of experiments that agree on the sign of change (robustness).

It is also of interest to understand how extreme precipitation and flood changes would vary with the seasonal variation of water availability. Because there is little land in the Southern Hemisphere mid-latitudes, the seasonal analysis is limited to the Northern Hemisphere mid-latitudes with a strong seasonal cycle of water availability. Similar to the relationships with the spatially-varying water availability over the globe, extreme precipitation changes have a clear connection with seasonal water availability (Fig. 4a). During the wet season when there is a moisture surplus, extreme precipitation increases get close to or exceeds the Clausius–Clapeyron rate, while the increase is smaller during the dry season. The extreme precipitation significantly and robustly increases in DJF (December–January–February), SON (September–October–November), MAM (March–April–May) and JJA (June–July–August) at the rates of 7.26, 6.70, 5.98 and 4.95%/K, respectively. A similar seasonal water availability dependence of changes is obtained for flood intensity (Fig. 4b). While small and insignificant flood intensity increases of 0.89 and 1.16%/K respectively are seen for JJA and MAM, the increase gets as large as 5.90 and 9.53%/K in SON and DJF, respectively.

### Response of precipitation and flood extremity to water availability

In order to explore the response of precipitation and flood extremity to the spatial and seasonal variations of water availability, all the analyses are repeated for less extreme precipitation and flood events with return periods ranging from 2 to 29 years. The less the extreme precipitation, the stronger the relationship of the changes with water availability (Fig. S13). The slope of changes with spatial water availability (from humid to arid regions) decays faster with precipitation extremity than that with seasonal water availability (from DJF to JJA). Similar to extreme precipitation, a weaker change relationship with spatial water availability is found for rarer flood events. The relationship of flood changes with seasonal water availability is, however, independent of flood extremity.

**Figure 5:**
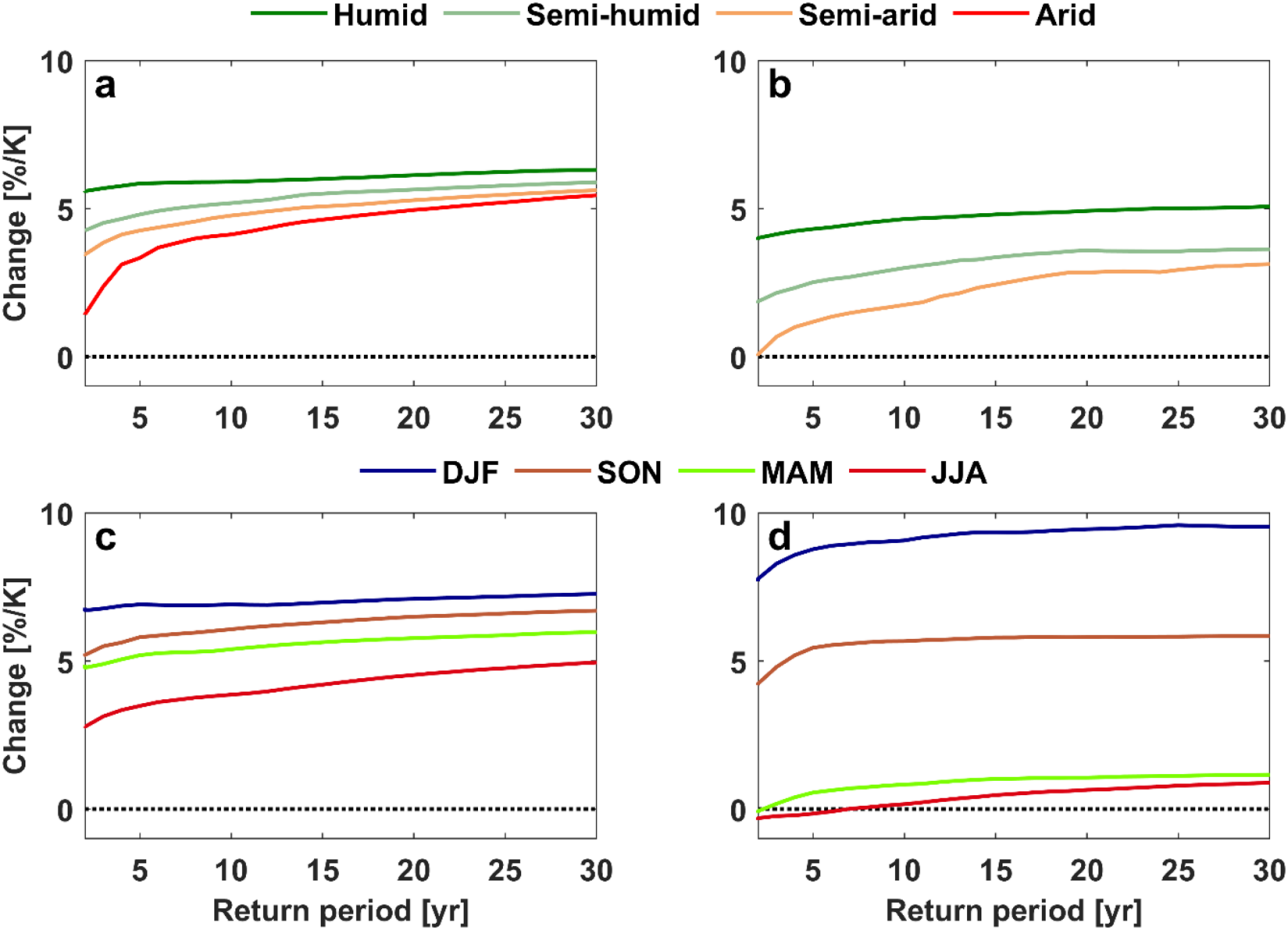
Intensity changes in (**a**,**c**) extreme precipitation and (**b**,**d**) flood events with return periods ranging between 2 and 30 years per (**a**,**b**) climate regime and (**c**,**d**) season based on multi-model ensemble median. The changes per seasons are computed for the Northern Hemisphere mid-latitudes.

Extreme precipitation and flood changes in different climate regions converge for more extreme events, due to a faster increment of the increases with event extremity in drier climates (Fig. 5). The thermodynamic factors play the main role for more extreme precipitation changes^44^, while for less extreme events dynamic factors are also responsible for regional precipitation changes which may weaken the thermodynamic effect^4–6^. In a similar manner, extreme precipitation changes in different seasons converge for more extreme events, because of a faster increment of the increases with event extremity in drier seasons (Fig. 5).

In terms of the area coverage of increasing signals, while the percentage of wet land areas (humid and semi-humid regions) with an increasing intensity signal of extreme precipitation remain almost constant when events become more extreme, the percentage area noticeably increases with event extremity in drier regions (e.g., from 51.9% for 1-in-2-year events to 98.7% for 1-in-30-year events in arid regions) (Fig. S14). Likewise, the percentage area of drier regions with an increasing flood intensity signal rises larger with event extremity: 20% area increase from 1-in-2-year events to 1-in-30-year events in semi-arid regions versus 11% and 5% area increases in semi-humid and humid regions, respectively (Fig. S14). A similar pattern is found in the seasonal analysis where a larger increment of the land area of increasing signals with event extremity is seen in dry season compared to wet season: from 83% (71%) for 1-in-2-year extreme precipitation to 96% (77%) for 1-in-30-year extreme precipitation (flood) in DJF as opposed to from 73% (42%) for 1-in-2-year extreme precipitation to 95% (56%) for 1-in-30-year extreme precipitation (flood) in JJA (Fig. S15). For more extreme events, the changes in flood intensities better follow the pattern of the extreme precipitation changes. This is because, for more extreme events, flood timing is more likely to correspond to rainfall timing, while for less extreme events it is more influenced by soil moisture timing^45^.

## Conclusions

The results of this study suggest that changes in flood and extreme precipitation intensities in response to global warming are significant and robust when aggregated over different climate regions. Regionalization of the changes decreases the large noise of extreme events at local scale, leading to more robust results. The increase in extreme precipitation and the expected decrease in total precipitation in dry regions^46,47^ supports “it never rains, but it pours” pattern^48^ in these regions. The results show a clear connection of the flood and extreme precipitation changes with spatial and seasonal water availability, pointing to a larger increase for the regions and seasons with higher water (moisture) availability. Limited climatological water availability in dry environments may offset extreme precipitation increases, while in water-abundance conditions amplified atmospheric moisture convergence can intensify the effects of extreme precipitation^13^. This suggests that attention should be paid not only on how much water the atmosphere can hold, but on how much water is available in the first place.

The flood changes in this paper are computed using two hazard quantification methods based a multi-model ensemble of 20 members including four GCMs and five IMs. Although it covers some important sources of uncertainty especially possible underestimation of flood changes from a single hydrologic model^23^, there exist other uncertainty sources related to the choice of hydrological model parameters, bias-correction approaches and downscaling methods which may further expand the uncertainty range. Moreover, the subset of four GCMs used as the climate forcing in the ISIMIP IMs may underestimate the full uncertainty in extreme precipitation projections from the CMIP5 ensemble (Fig. S16). The uncertainty associated with the hazard quantification methodology, less quantified in previous studies, is particularly important in arid regions and needs to be included in future climate change assessments on extreme events.

## Methods

### Data overview

Daily precipitation simulations from 24 Coupled Model Intercomparison Project Phase 5 (CMIP5^33^) GCMs for the historical period 1971–2000 and the future period 2070–2099 forced by Representative Concentration Pathway (RCP) RCP8.5 scenario are utilized: ACCESS1-0, ACCESS1-3, CanESM2, CMCC-CESM, CMCC-CM, CMCC-CMS, CNRM-CM5, CSIRO-Mk3-6-0, GFDL-CM3, GFDL-ESM2G, GFDL-ESM2M, HadGEM2-AO, HadGEM2-CC, Had-GEM2-ES, INMCM4, IPSL-CM5A-LR, IPSL-CM5AMR, IPSL-CM5B-LR, MIROC5, MIROC-ESM, MIROC-ESM-CHEM, MPI-ESM-LR, MPI-ESM-MR, MRI-CGCM3. The first initial condition run (r1) of each CMIP5 GCM is considered to ensure equal weighting of all models in a multi-model ensemble median. Monthly maximum, mean and minimum temperature data for the historical and RCP8.5 simulations from the same GCMs are also used for climate classification and global warming estimation. The selection of the CMIP5 GCMs is made based on the availability of daily precipitation and monthly temperature data for historical and RCP8.5 simulations. The simulations of the 24 CMIP5 GCMs used in this study are of varying spatial resolutions (0.75°–3.75°). They are therefore resampled to a common 0.5° × 0.5° grid to match the spatial resolution of the discharge data. In order to minimize errors in the calculation of extreme precipitation changes^49^, the changes are first computed on native model grids and then are interpolated to the common grid using the bilinear interpolation method. It was shown that the resampling results are not sensitive to the choice of the interpolation method and of the common grid size^9^. The extreme precipitation analyses are restricted to all land grid cells where the impact of changes is predominantly felt.

In the Inter-Sectoral Impact Model Intercomparison Project (ISIMIP^34^) framework, four of the CMIP5 GCMs (GFDL-ESM2M, HadGEM2-ES, IPSL-CM5A-LR and MIROC5) were downscaled by a trend-preserving method^50^ and used as the climatic forcing to simulate daily discharge. Global daily discharge simulations from five impact models (IMs) including three global hydrological models (H08^51^, MATSIRO^52^ and WaterGAP2^53^), one global land surface model (CLM4.5^54^) and one dynamic global vegetation model (LPJmL^55^) with 0.5° spatial resolution for the historical period 1971–2000 and the future period 2070–2099 under RCP8.5 scenario are used. All IMs were setup using the same soil, land cover and morphologic data such that discrepancies among them is only due to their different process representations. The simulations with a varying land use and other human influences over the historical period (“histsoc” experiment) and then fixed at 2005 levels for the future period are used for all the IMs except CLM4.5 which uses fixed year-2005 socioeconomic conditions (“2005soc” experiment) for both historical and future periods. The preliminary analysis shows that the influence of socioeconomic scenarios on flood intensity changes is minor (Fig. S1). Grid cells with annual maxima close to 0 m^3^ s^−1^ of the historical period are screened out for the further analysis, due to insufficient data for distribution fitting and smaller importance for flood analysis. The high latitude area in the Southern Hemisphere (> 60°S) which it is almost uninhabited and not subject to flooding is also excluded.

### Quantification of climate change signals

A 30-year return level of precipitation and river flow (average occurrence of once every 30 years) at each grid cell is used as the indicator of extreme precipitation and flooding. To this end, annual maxima time series of extreme precipitation and flow for each grid cell are derived for both historical and future periods. The generalized extreme value distribution (GEV) is fitted to the annual maxima series on the native grids of each model. The GEV distribution is characterized by three parameters including location (µ; describing the center of distribution), scale (σ; describing the deviation around the mean) and shape ($\upxi$; describing the tail behavior of the distribution) of the distribution. According to the shape parameter, three extreme distributions are defined as Fréchet, Gumbel and Weilbull corresponding to $\upxi$ > 0, $\upxi$ = 0 and $\upxi$ < 0, respectively. To quantify the uncertainty associated to the hazard quantification method, the extreme precipitation and flood hazards are also determined using the peak-over-threshold (POT) method with a Generalized Pareto Distribution (GPD). The GPD distribution is also characterized by the location, scale and shape parameters and it leads to Pareto, Exponential and Beta distributions for $\upxi$ > 0, $\upxi$ = 0 and $\upxi$ < 0, respectively. The GEV and GPD parameters are estimated using the maximum likelihood method.

Changes in extreme precipitation and flood intensities are defined as the ratio between the intensities of the end twenty-first century (2070–2099) and the end twenty century (1971–2000). To accounts for the effect of different climate sensitivities of the CMIP5 GCMs, extreme precipitation and flood changes of individual GCMs are scaled by their changes in global average surface air temperature (Fig. S2) to derive units of %/K. Change in global average surface air temperature is calculated by comparing the 30-year global average annual temperature between the historical period 1971–2000 and the future period 2070–2099 under RCP8.5 scenario.

### Association of changes with water availability

To investigate the relationships of the changes in extreme precipitation and flood intensities with climatological water availability, the climatological water availability of each grid of the CMIP5 models for historical (1971–2000) and future (2070–2099) periods is determined based on the aridity index (AI). AI is calculated as the ratio between potential evapotranspiration (PET; water demand) and precipitation (P; water supply): AI = PET/P. The grids with AI < 1 are classified as humid, 1 ≤ AI < 2 as semi-humid, 2 ≤ AI < 5 as semi-arid and AI ≥ 5 as arid^56,57^. PET is computed by the Hargreaves–Samani method^58^ which effectively incorporates solar radiation by its indirect estimation from minimum and maximum temperatures. The use of both minimum and maximum temperature avoids the PET overestimation in dry and hot climates by methods based on only mean temperature such as the Thornthwaite method^28,59,60^. To investigate the possible impact of climate change on climate regimes (aridity classifications), the change in the area of each class (% of total terrestrial land area) is calculated. Once the spatial distribution of the global climate regimes is acquired, the median change of extreme precipitation and flood intensity for each climate regime is determined.

The significance of the changes is assessed per climate regime using the signal-to-noise (S2N) ratio. In this way, the large internal variability of extremes at local scale^61,62^ can be decreased, leading to more robust regional results^63^. For extreme precipitation intensity, S2N is computed by dividing the ensemble median of changes across the 24 GCMs by the standard deviation of the multi-model changes. For flood intensity, S2N is calculated first across all GCMs for each individual IM per climate regime and then the median across all IMs is considered as S2N per climate regime. A signal is significant at 90% and 95% confidence levels when S2N is larger 1.64 and 1.96, respectively. A similar procedure is applied for the robustness of the changes in extreme precipitation and flood intensity where change for each climate regime is considered robust when at least 75% of experiments agree on the sign of the change.

### Assessment of uncertainty sources

The uncertainty in the projected changes in the intensity of extreme precipitation and flood events is also quantified for each model grid. The extreme precipitation and flood ensembles include 48 (2 methods × 24 GCMs) and 40 (2 methods × 4 GCMs × 5 IMs) experiments, respectively. The total uncertainty of flood changes expressed as the coefficient of variation (CV) of the changes across the full ensemble is decomposed into hazard quantification method, GCM and IM uncertainties, while that of extreme precipitation changes is split into hazard quantification method and GCM uncertainties. The uncertainty for the components with a larger sample size tends to be larger than those with a smaller sample size^64^. To limit this bias, the variance decomposition-same sample size (VD-SSS^9^) method is employed for the uncertainty quantification of the GCM and IM components with larger sizes, while the conventional variance decomposition (VD) method is applied for the hazard quantification method uncertainty. The VD-SSS method uses an iterative sampling-theory based bootstrapping procedure where a sample of size n (equal to the smallest sample size among the uncertainty components) is first drawn randomly from the full population of size N (e.g., 24 for GCMs). The CV across the bootstrap samples is then estimated. This procedure is repeated a large number of times (1,000 iterations in this study) and the median of the empirical bootstrap distribution of sample CV denotes the uncertainty. To understand how uncertainty differs between climate regions, both total and fractional uncertainties are computed per climate regime.

## Supplementary information

Supplementary Information.

## References

[1] Allen MR, Ingram WJ (2002). Constraints on future changes in climate and the hydrologic cycle. Nature.

[2] Trenberth KE, Dai A, Rasmussen RM, Parsons DB (2003). The changing character of precipitation. Bull. Am. Meteorol. Soc..

[3] Ingram W (2016). Extreme precipitation: increases all round. Nat. Clim. Change.

[4] Scheff J, Frierson DMW (2012). Robust future precipitation declines in CMIP5 largely reflect the poleward expansion of model subtropical dry zones. Geophys. Res. Lett..

[5] Pfahl S, O’Gorman PA, Fischer EM (2017). Understanding the regional pattern of projected future changes in extreme precipitation. Nat. Clim. Change.

[6] Norris J, Chen G, Neelin JD (2019). Thermodynamic versus dynamic controls on extreme precipitation in a warming climate from the community earth system model large ensemble. J. Clim..

[7] Li C (2019). Larger increases in more extreme local precipitation events as climate warms. Geophys. Res. Lett..

[8] Zhang X, Zwiers FW, Li G, Wan H, Cannon AJ (2017). Complexity in estimating past and future extreme short-duration rainfall. Nat. Geosci..

[9] Tabari H, Hosseinzadehtalaei P, AghaKouchak A, Willems P (2019). Latitudinal heterogeneity and hotspots of uncertainty in projected extreme precipitation. Environ. Res. Lett..

[10] Roderick TP, Wasko C, Sharma A (2019). Atmospheric moisture measurements explain increases in tropical rainfall extremes. Geophys. Res. Lett..

[11] Hardwick Jones R, Westra S, Sharma A (2010). Observed relationships between extreme sub-daily precipitation, surface temperature, and relative humidity. Geophys. Res. Lett..

[12] Drobinski P, Alonzo B, Bastin S, Da Silva N, Muller C (2016). Scaling of precipitation extremes with temperature in the French Mediterranean region: What explains the hook shape?. J. Geophys. Res. Atmos..

[13] Held IM, Soden BJ (2006). Robust responses of the hydrological cycle to global warming. J. Clim..

[14] Donat MG, Lowry AL, Alexander LV, O’Gorman PA, Maher N (2016). More extreme precipitation in the world’s dry and wet regions. Nat. Clim. Change.

[15] Donat MG, Angélil O, Ukkola AM (2019). Intensification of precipitation extremes in the world’s humid and water-limited regions. Environ. Res. Lett..

[16] Pendergrass AG (2018). What precipitation is extreme?. Science.

[17] Myhre G (2019). Frequency of extreme precipitation increases extensively with event rareness under global warming. Sci. Rep..

[18] Berg P, Haerter JO, Thejll P, Piani C, Hagemann S, Christensen JH (2009). Seasonal characteristics of the relationship between daily precipitation intensity and surface temperature. J. Geophys. Res..

[19] Sharma A, Wasko C, Lettenmaier DP (2018). If precipitation extremes are increasing, why aren't floods?. Water Resour. Res..

[20] Blöschl G (2017). Changing climate shifts timing of European floods. Science.

[21] Hamlet AF, Lettenmaier DP (2007). Effects of 20th century warming and climate variability on flood risk in the western U.S. Water Resour. Res..

[22] Hirabayashi Y (2013). Global flood risk under climate change. Nat. Clim. Change.

[23] Dankers R (2014). First look at changes in flood hazard in the Inter-Sectoral Impact Model Intercomparison Project ensemble. Proc. Natl Acad. Sci. USA.

[24] Arnell NW, Gosling SN (2016). The impacts of climate change on river flood risk at the global scale. Clim. Change.

[25] Asadieh B, Krakauer NY (2017). Global change in streamflow extremes under climate change over the 21st century. Hydrol. Earth Syst. Sci..

[26] Kundzewicz ZW (2017). Differences in flood hazard projections in Europe—their causes and consequences for decision making. Hydrol. Sci. J..

[27] Wolock DM, McCabe GJ (1999). Explaining spatial variability in mean annual runoff in the conterminous United States. Clim. Res..

[28] Girvetz EH, Zganjar C (2014). Dissecting indices of aridity for assessing the impacts of global climate change. Clim. Change.

[29] Kumar S, Zwiers F, Dirmeyer PA, Lawrence DM, Shrestha R, Werner AT (2016). Terrestrial contribution to the heterogeneity in hydrological changes under global warming. Water Resour. Res..

[30] Koster RD, Suarez MJ (1999). A simple framework for examining the interannual variability of land surface moisture fluxes. J. Clim..

[31] Sankarasubramanian A, Vogel RM (2002). Annual hydroclimatology of the United States. Water Resour. Res..

[32] Zhang L, Potter N, Hickel K, Zhang Y, Shao Q (2008). Water balance modeling over variable time scales based on the Budyko framework—model development and testing. J. Hydrol..

[33] Taylor KE, Stouffer RJ, Meehl GA (2012). An overview of CMIP5 and the experiment design. Bull. Am. Meteorol. Soc..

[34] Warszawski L, Frieler K, Huber V, Piontek F, Serdeczny O, Schewe J (2014). The Inter-Sectoral Impact Model Intercomparison Project (ISI-MIP): project framework. Proc. Natl Acad. Sci. USA.

[35] Kharin VV, Flato GM, Zhang X, Gillett NP, Zwiers F, Anderson KJ (2018). Risks from climate extremes change differently from 1.5 C to 2.0 C depending on rarity. Earth's Future.

[36] Kharin VV, Zwiers FW, Zhang X, Wehner M (2013). Changes in temperature and precipitation extremes in the CMIP5 ensemble. Clim. Change.

[37] Bador M, Donat MG, Geoffroy O, Alexander LV (2018). Assessing the robustness of future extreme precipitation intensification in the CMIP5 ensemble. J. Climat..

[38] Thober S (2018). Multi-model ensemble projections of European river floods and high flows at 1.5, 2, and 3 degrees global warming. Environ. Res. Lett..

[39] Rojas R, Feyen L, Bianchi A, Dosio A (2012). Assessment of future flood hazard in Europe using a large ensemble of bias-corrected regional climate simulations. J. Geophys. Res. Atmos..

[40] Alfieri L, Burek P, Feyen L, Forzieri G (2015). Global warming increases the frequency of river floods in Europe. Hydrol. Earth Syst. Sci..

[41] Wasko C, Nathan R (2019). Influence of changes in rainfall and soil moisture on trends in flooding. J. Hydrol..

[42] Hagemann S (2013). Climate change impact on available water resources obtained using multiple global climate and hydrology models. Earth Syst. Dyn..

[43] Giuntoli I (2015). Future hydrological extremes: the uncertainty from multiple global climate and global hydrological models. Earth Syst. Dyn..

[44] Emori S, Brown SJ (2005). Dynamic and thermodynamic changes in mean and extreme precipitation under changed climate. Geophys. Res. Lett..

[45] Wasko C, Nathan R, Peel MC (2020). Changes in antecedent soil moisture modulate flood seasonality in a changing climate. Water Resour. Res..

[46] Marvel K, Bonfils C (2013). Identifying external influences on global precipitation. Proc. Natl Acad. Sci. USA.

[47] Tabari H, Willems P (2018). More prolonged droughts by the end of the century in the Middle East. Environ. Res. Lett..

[48] Trenberth KE (2011). Changes in precipitation with climate change. Clim. Res..

[49] Diaconescu EP, Gachon P, Laprise R (2015). On the remapping procedure of daily precipitation statistics and indices used in regional climate model evaluation. J. Hydrometeor..

[50] Hempel S, Frieler K, Warszawski L, Schewe J, Piontek F (2013). A trend-preserving bias correction—The ISI-MIP approach. Earth Syst. Dyn..

[51] Hanasaki N (2008). An integrated model for the assessment of global water resources—Part 1: Model description and input meteorological forcing. Hydrol. Earth Syst. Sci..

[52] Takata K, Emori S, Watanabe T (2003). Development of the minimal advanced treatments of surface interaction and runoff. Glob. Planet. Change.

[53] Mueller Schmied H (2016). Variations of global and continental water balance components as impacted by climate forcing uncertainty and human water use. Hydrol. Earth Syst. Sci..

[54] Thiery W, Davin EL, Lawrence DM, Hirsch AL, Hauser M, Seneviratne SI (2017). Present-day irrigation mitigates heat extremes. J. Geophys. Res. Atmos..

[55] Bondeau A (2007). Modelling the role of agriculture for the 20th century global terrestrial carbon balance. Glob. Chang. Biol..

[56] UNEP (1997). World Atlas of Desertification.

[57] Ukkola AM, Prentice IC, Keenan TF, van Dijk AIJM, Viney NR, Myneni RB, Bi J (2016). Reduced streamflow in waterstressed climates consistent with CO2 effects on vegetation. Nat. Clim. Change.

[58] Hargreaves GH, Samani ZA (1985). Reference crop evapotranspiration from temperature. Appl. Eng. Agric..

[59] Shahidian S, Serralheiro R, Serrano J, Teixeira J, Haie N, Santos F, Irmark A (2012). Hargreaves and other reduced-set methods for calculating evapotranspiration. Evapotranspiration: Remote Sensing and Modeling.

[60] Spinoni J (2020). Future global meteorological drought hotspots: a study based on CORDEX data. J. Clim..

[61] Fischer EM, Knutti R (2016). Observed heavy precipitation increase confirms theory and early models. Nat. Clim. Change.

[62] Fischer EM, Sedláček J, Hawkins E, Knutti R (2014). Models agree on forced response pattern of precipitation and temperature extremes. Geophys. Res. Lett..

[63] Hosseinzadehtalaei P, Tabari H, Willems P (2019). Regionalization of anthropogenically forced changes in 3 hourly extreme precipitation over Europe. Environ. Res. Lett..

[64] Hosseinzadehtalaei P, Tabari H, Willems P (2017). Uncertainty assessment for climate change impact on intense precipitation: how many model runs do we need?. Int. J. Climatol..

[65] MATLAB and Mapping Toolbox Release 2019a. Mapping Toolbox User’s Guide—map_ug.pdf. https://www.mathworks.com/products/mapping.html. Accessed 27 Mar 2020 (2019).

