# Author Correction: Climate change impact on flood and extreme precipitation increases with water availability

**DOI:** 10.1038/s41598-020-74038-4

**Published:** 2020-10-07

**Authors:** Hossein Tabari

**Affiliations:** Department of Civil Engineering, KU Leuven, Leuven, Belgium

Correction to: *Scientific Reports* https://doi.org/10.1038/s41598-020-70816-2, published online 13 August 2020

The original version of this Article contained a typographical error in the Abstract.

“Results show an intensification of extreme precipitation and flood events over all climate regions which increases as water availability increases from wet to dry regions.”

now reads:

“Results show an intensification of extreme precipitation and flood events over all climate regions which increases as water availability increases from dry to wet regions.”

This error has now been corrected in the PDF and HTML versions of the Article.

