# Supplementary material for: Climate change impact on flood and extreme precipitation increases with water availability
Source: Sci Rep. 2020 Aug 13;10:13768. doi: 10.1038/s41598-020-70816-2 (PMC7426818; doi:10.1038/s41598-020-70816-2)
Supplement: Supplementary file 1 — Supplementary Information. [file 41598_2020_70816_MOESM1_ESM.pdf]

Supplementary information for

Climate change impact on flood and extreme precipitation increases with water availability

Hossein Tabari

KU Leuven, Department of Civil Engineering, Belgium (hossein.tabari@kuleuven.be)

This PDF file includes:

Texts S1 to S4

Figs. S1 to S16

Text S1. Sensitivity of flood changes to socioeconomic scenario. The simulations with a varying land use and other human influences over the historical period ("histsoc" experiment) and then fixed at 2005 levels for the future period are used for all the employed global impact models (IMs) except CLM4.5 which uses fixed year-2005 socioeconomic conditions ("2005soc" experiment) for both historical and future periods. To analyze the sensitivity of flood changes to the socioeconomic scenario, the projected changes in 1-in-30-year flood intensity between histsoc and 2005soc socioeconomic scenarios of historical simulations of H08 model are compared. The results in Fig. S1 show almost identical spatial distribution of changes for the two cases, implying the negligible influence of the socioeconomic scenarios of historical simulations on the spatial distribution of the changes. In terms of change magnitude, the difference between two socioeconomic scenarios is also minor. As expected, the historical socioeconomic scenario of the simulations leads to slightly larger changes compared to a fixed year-2005 socioeconomic scenario; however, the difference is less than 0.06 %/K.

Text S2. Estimation of global warming. Global warming is calculated by comparing the 30-year global average annual temperature between the historical period 1971–2000 and the future period 2070–2099 under RCP8.5 scenario. The global warming estimations for the end of the 21st century from the current climate are presented in Fig. S2. The global warming for the 24 CMIP5 GCMs ranges from 3.4 to 6.2 K, with an ensemble median of 5.0 K. Future global warming estimates from the four GCMs used as the climate forcing of ISIMIP discharge simulations can capture the full range of uncertainty in the estimates from the ensemble of the 24 CMIP5 GCMs. The global warming from these four GCMs is within the same range of 3.4-6.2 K, with a median of 4.9 K.

Text S3. Bias of using the ensemble median mask of climate regimes. Because of the discrepancy among GCMs on the geographical distribution of climate regimes, the relationships of extreme precipitation and flood changes with water availability is investigated based on model-specific masks of climate regimes. In order to examine the bias of using the ensemble median mask of climate regimes instead of individual model masks, the changes in extreme precipitation and flood intensities are calculated based the ensemble median mask of climate regimes. The bias is considered as the deviation from the results derived from individual model masks. As shown in Figs. S11a and S12a, the bias in the computed changes is large for some models. For extreme precipitation (Fig. S11a), the bias increases as water availability decreases. IPSL models show the largest bias in changes for semi-arid and semi-humid regions: 2.7 and 2.4 %/K for IPSL-CM5A-LR, 3.9 and 2.3 %/K for IPSL-CM5A-MR and 2.5 and 1.8 %/K for IPSL-CM5B-LR for semi-arid and semi-humid regions, respectively. The bias of 2.5 %/K for MIROC GCM for the extreme precipitation changes in arid regions is also noticeable. As for flood changes (Fig. S12a), the bias also increases with decreasing water availability, accounting for an median bias of 0.69, 0.89 and 1.13 %/K for humid, semi-humid and semi-arid regions, respectively. The global impact models follow the bias of their forcing GCMs such that the impact models forced by IPSL-CM5A-LR have the largest bias: a median bias of 2.4 %/K for IPSL-CM5A-LR forced models versus <1 %/K for the models forced by the rest of GCMs.

Text S4. Bias of using masks of static climate regimes. As climate regions may change under future global warming, the relationships of extreme precipitation and flood changes with water availability is examined based on projected climate regions for the future. The bias of using static climate regimes is determined by comparing the changes in extreme precipitation and flood intensities based on static (current) and dynamic (future) climate regions masks. For both extreme precipitation and flood changes, the bias of static climate regime masks is less than that of the ensemble median mask. Fig. 11b shows that the bias in extreme precipitation changes generally increases from humid to arid climate and bias of > 1 %/K is seen in arid and semi-arid climate regimes. The largest bias for flood change is also found in the driest

climate (Fig. 12b). MIROC5 is the model with a large bias (Fig. 11b), leading to a large bias in the IMs forced by this model (Fig. 12b).

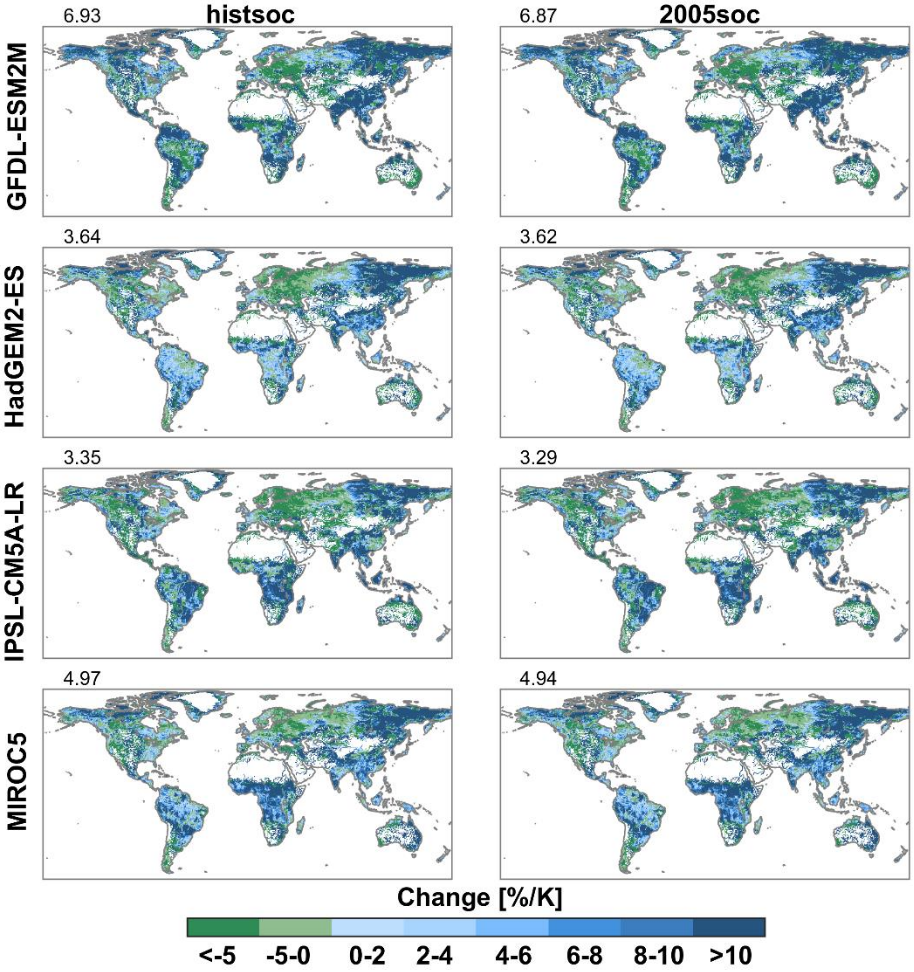

Figure S1. Comparison of projected changes (%) in 1-in-30-year flood intensity per K global warming in 2070–2099 under RCP8.5, compared with 1971–2000 between histsoc and 2005soc socioeconomic scenarios of historical simulations of H08 model. The numbers at the top indicate the global median of the change. The maps were generated using the MATLAB mapping toolbox[65] (URL-https://www.mathworks.com/products/mapping.html).

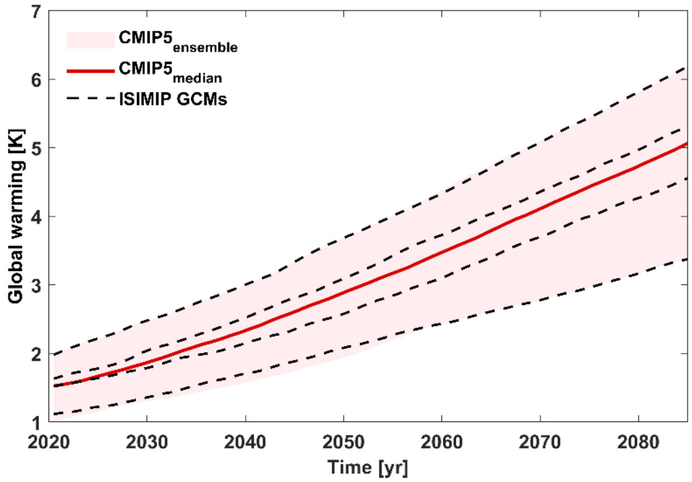

Figure S2. Global warming from the present to the future period using 24 CMIP5 GCMs with the 4 GCMs used in the ISIMIP project highlighted.

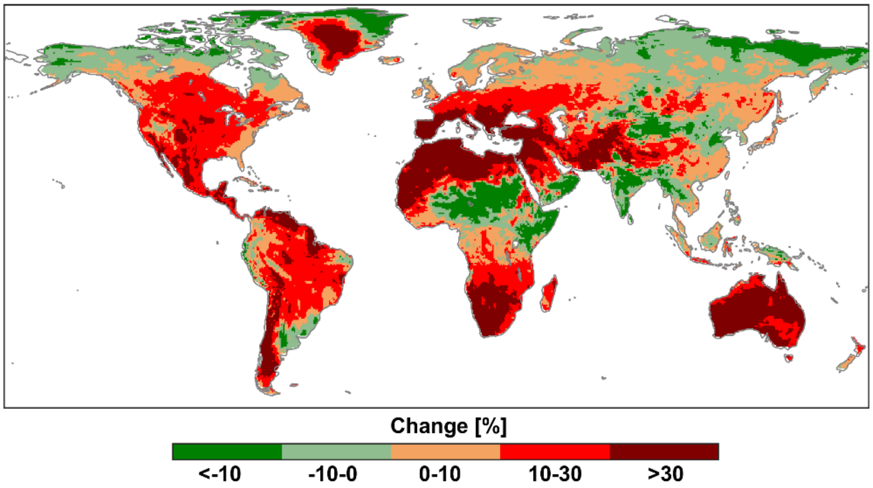

Figure S3. Spatial distribution of ensemble median changes in aridity index for the period 2070–2099 with respect to the reference 1971–2000. The map was generated using the MATLAB mapping toolbox[65] (URL-https://www.mathworks.com/products/mapping.html).

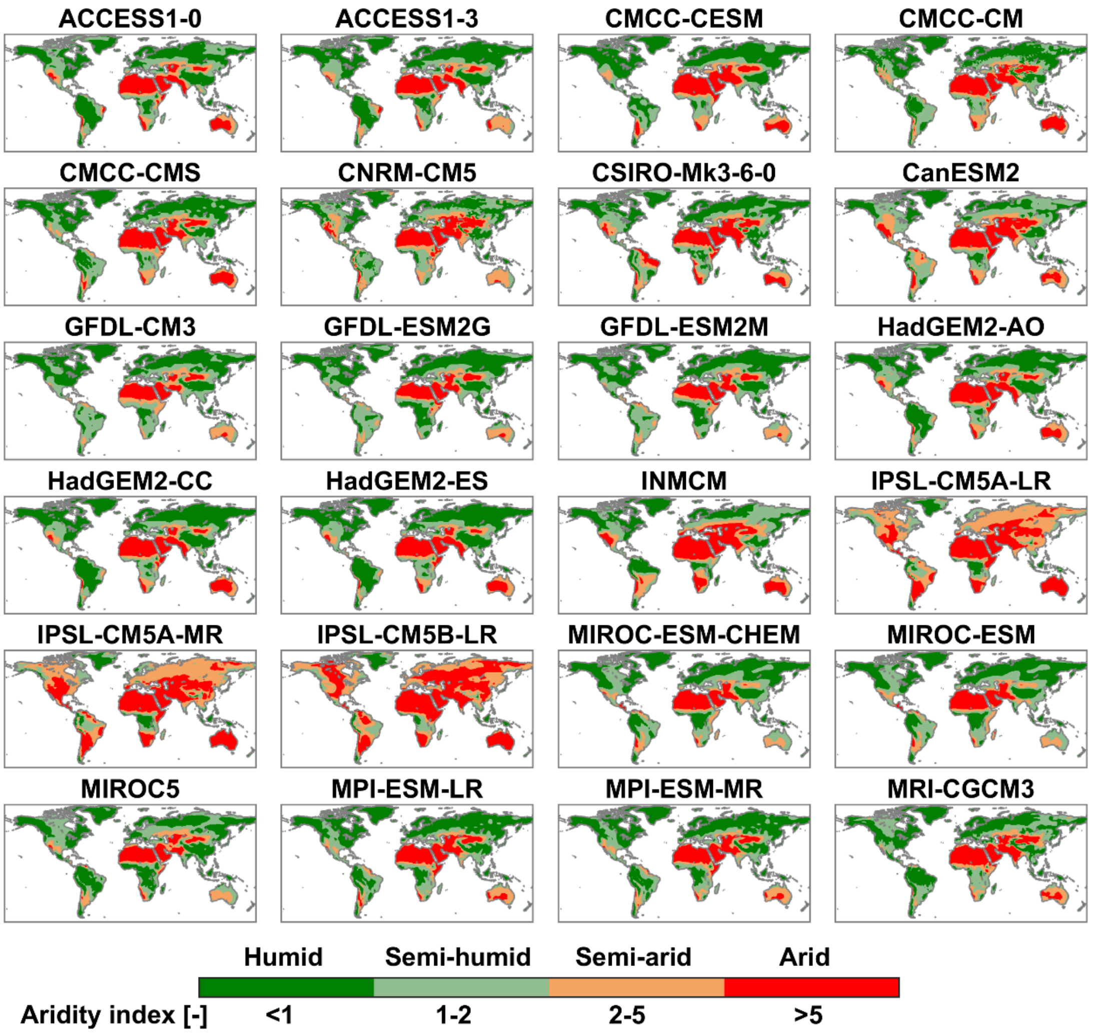

Figure S4. Spatial distribution of model-specific water availability masks based on the aridity index of the historical climate (1971–2000). The maps were generated using the MATLAB mapping toolbox[65] (URL- https://www.mathworks.com/products/mapping.html).

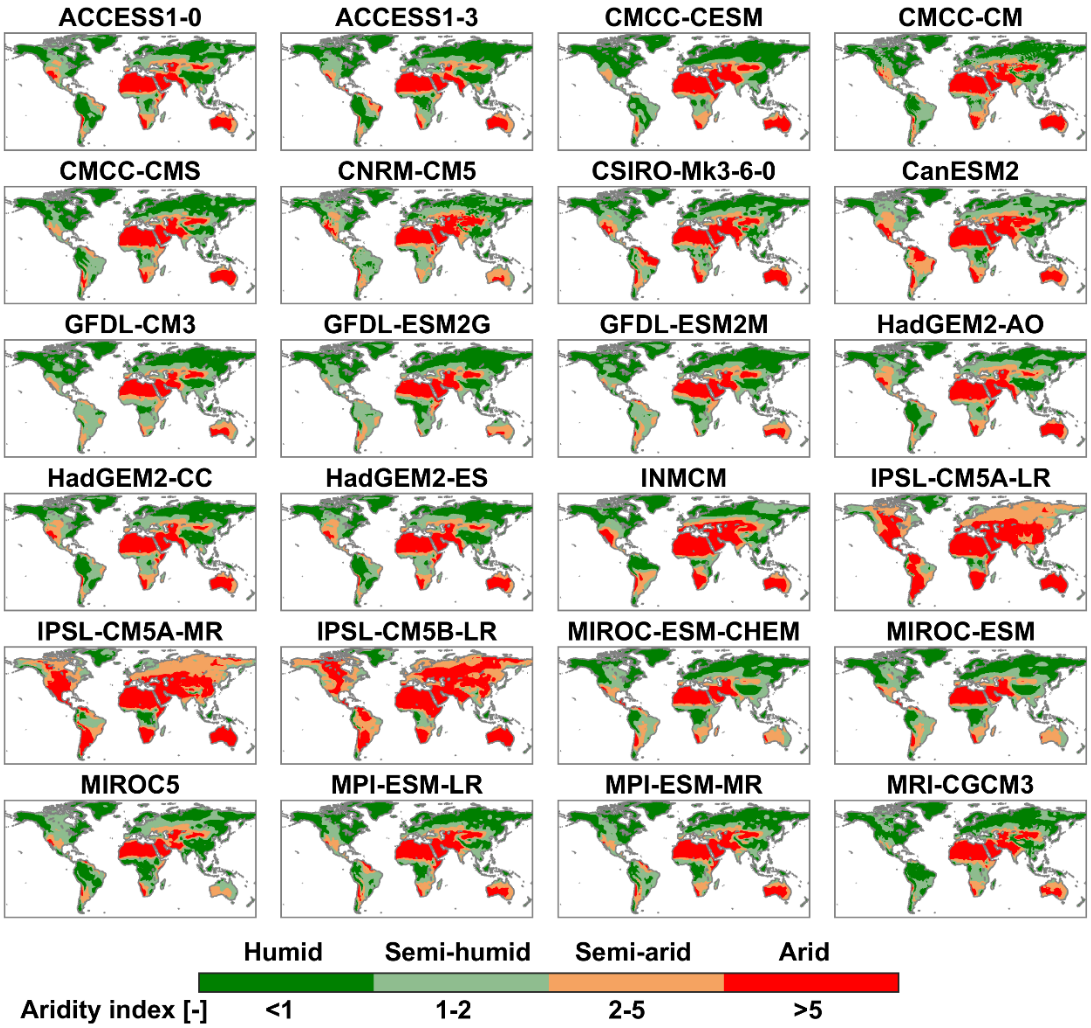

Figure S5. Spatial distribution of model-specific water availability masks based on the aridity index of the future climate (2070–2099). The maps were generated using the MATLAB mapping toolbox[65] (URL-https://www.mathworks.com/products/mapping.html).

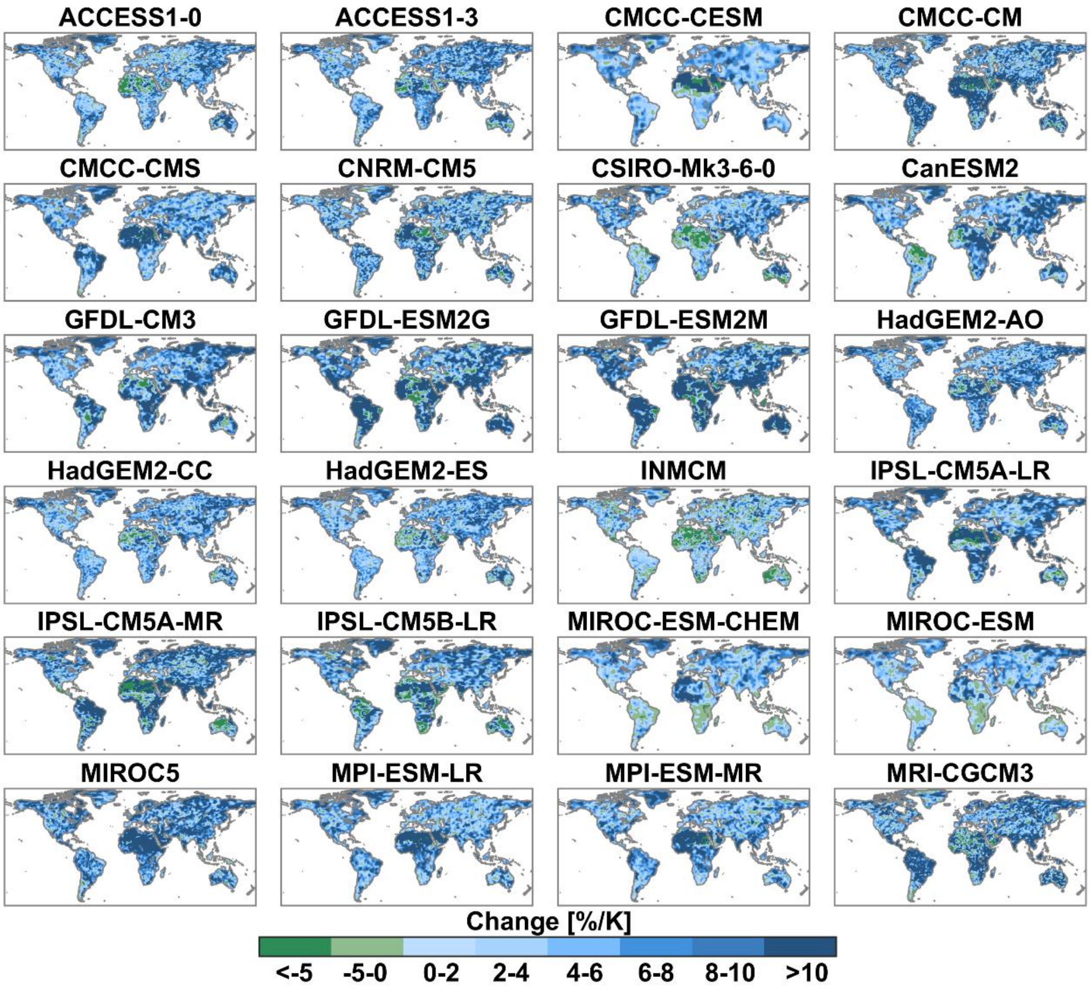

Figure S6. Spatial distribution of model-specific changes in 1-in-30-year extreme precipitation intensity per K global warming in 2070–2099 under RCP8.5, compared with 1971–2000. The maps were generated using the MATLAB mapping toolbox[65] (URL-https://www.mathworks.com/products/mapping.html).

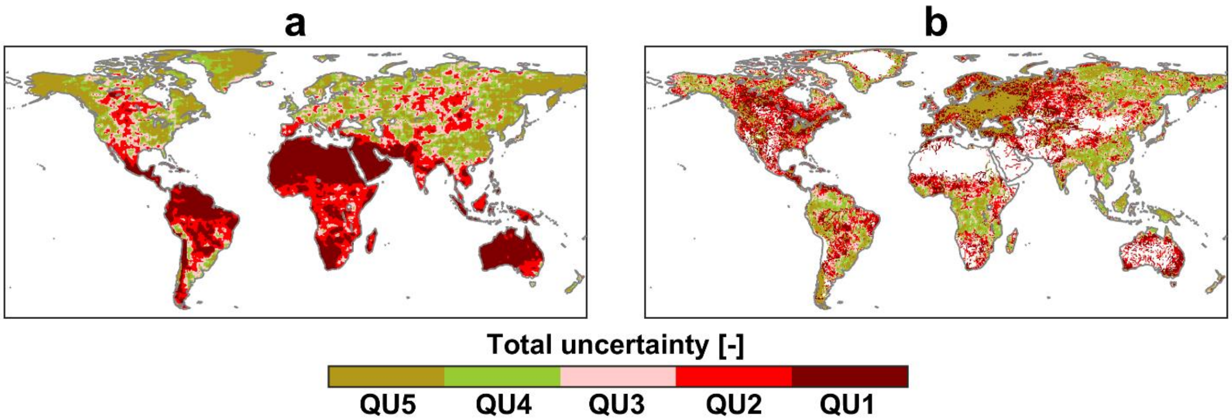

Figure S7. Total uncertainty of changes in 1-in-30-year (**a**) extreme precipitation and (**b**) flood intensity per K global warming in 2070–2099 under RCP8.5, compared with 1971–2000. Q1, Q2, Q3, Q4 and Q5 denote the first, second, third, fourth and fifth quintiles of the total uncertainty, respectively. In panel b, grid cells with annual maxima close to 0 $m^3 \cdot s^{-1}$ of the historical model period are screened out. The maps were generated using the MATLAB mapping toolbox[65] (URL-https://www.mathworks.com/products/mapping.html).

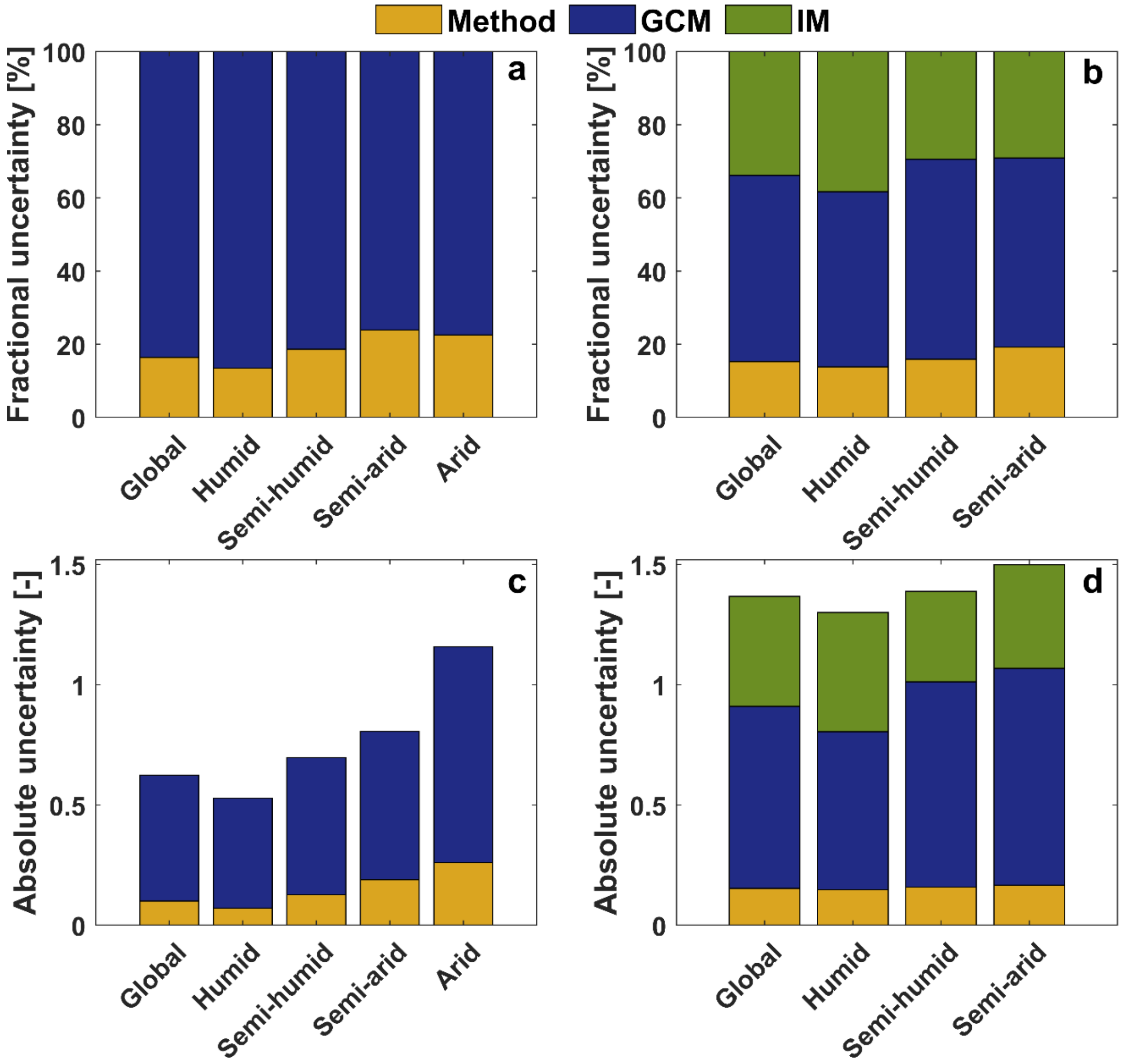

Figure S8. (**a**, **b**) Fractional and (**c**, **d**) absolute uncertainties of changes in 1-in-30-year (**a**, **c**) extreme precipitation and (**b**, **d**) flood intensity per K global warming in 2070–2099 under RCP8.5, compared with 1971–2000.

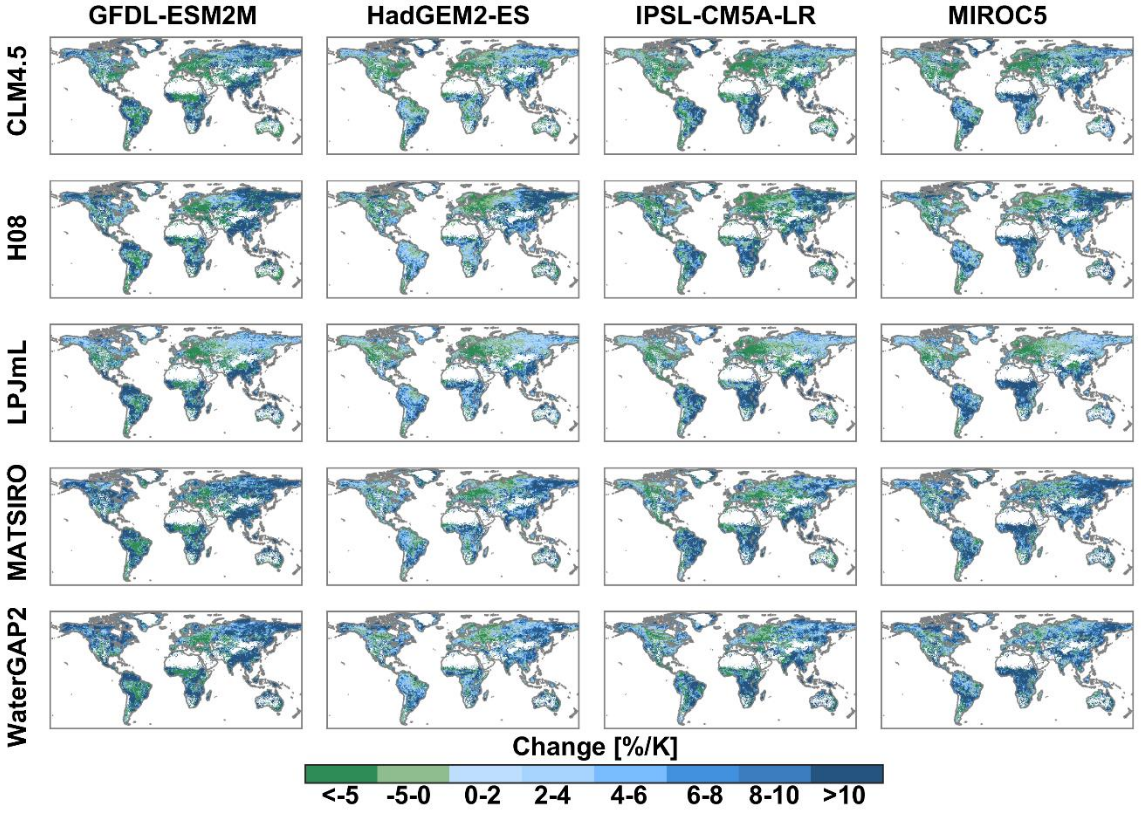

Figure S9. Spatial distribution of changes in 1-in-30-year flood intensity per K global warming for different IMs forced by different GCMs in 2070–2099 under RCP8.5, compared with 1971–2000. Grid cells with annual maxima close to 0 $m^3 \cdot s^{-1}$ of the historical model period are screened out. The maps were generated using the MATLAB mapping toolbox[65] (URL-https://www.mathworks.com/products/mapping.html).

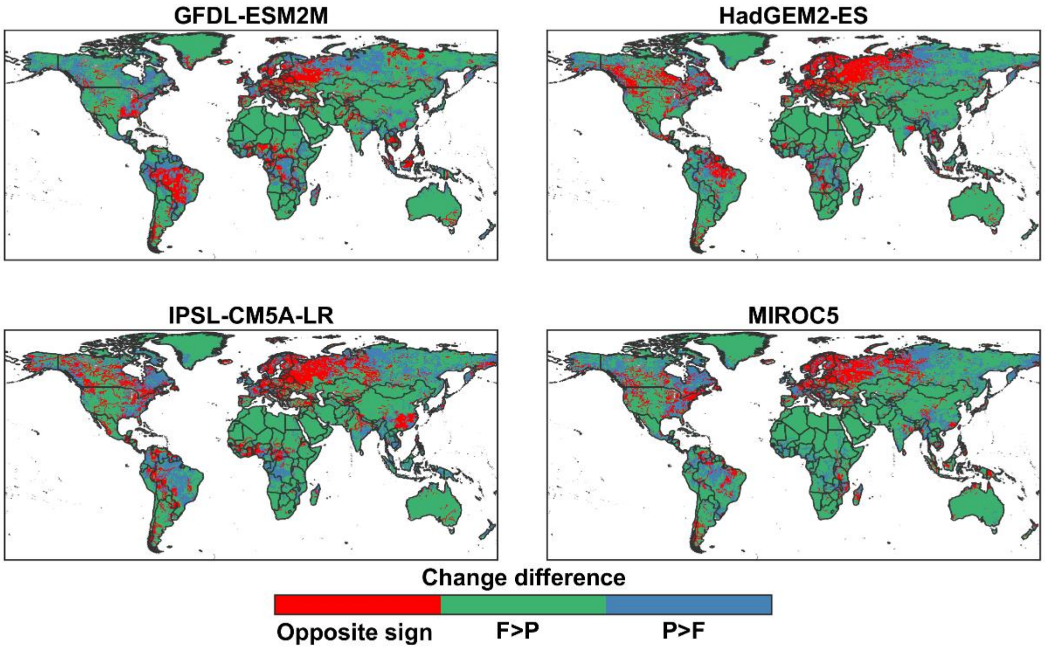

Figure S10. Comparison of changes in extreme precipitation (P) and flood (F) intensities per K global warming in 2070–2099 under RCP8.5, compared with 1971–2000. The maps were generated using the MATLAB mapping toolbox[65] (URL-https://www.mathworks.com/products/mapping.html).

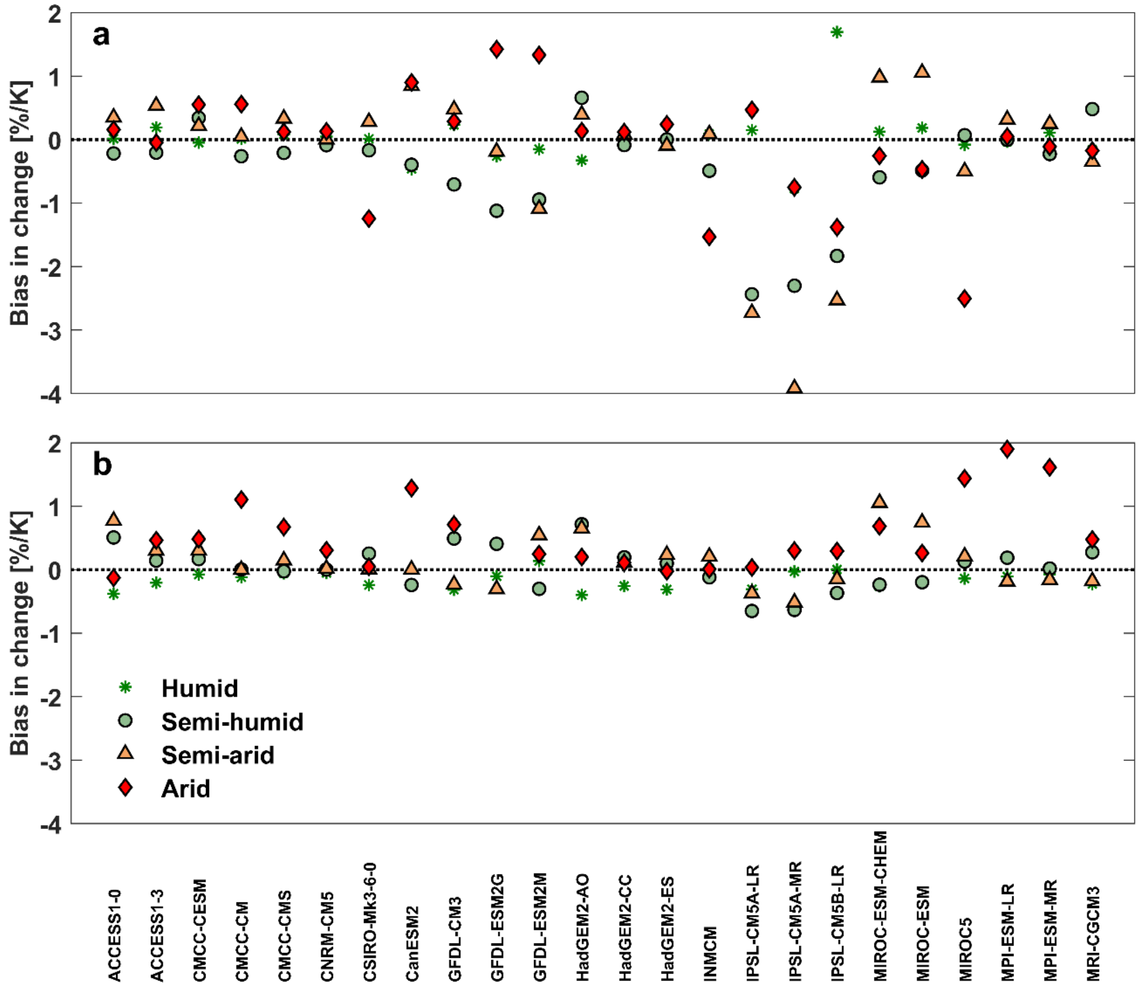

Figure S11. Bias in extreme precipitation intensity changes by using (**a**) median ensemble mask and (**b**) static climate regime masks.

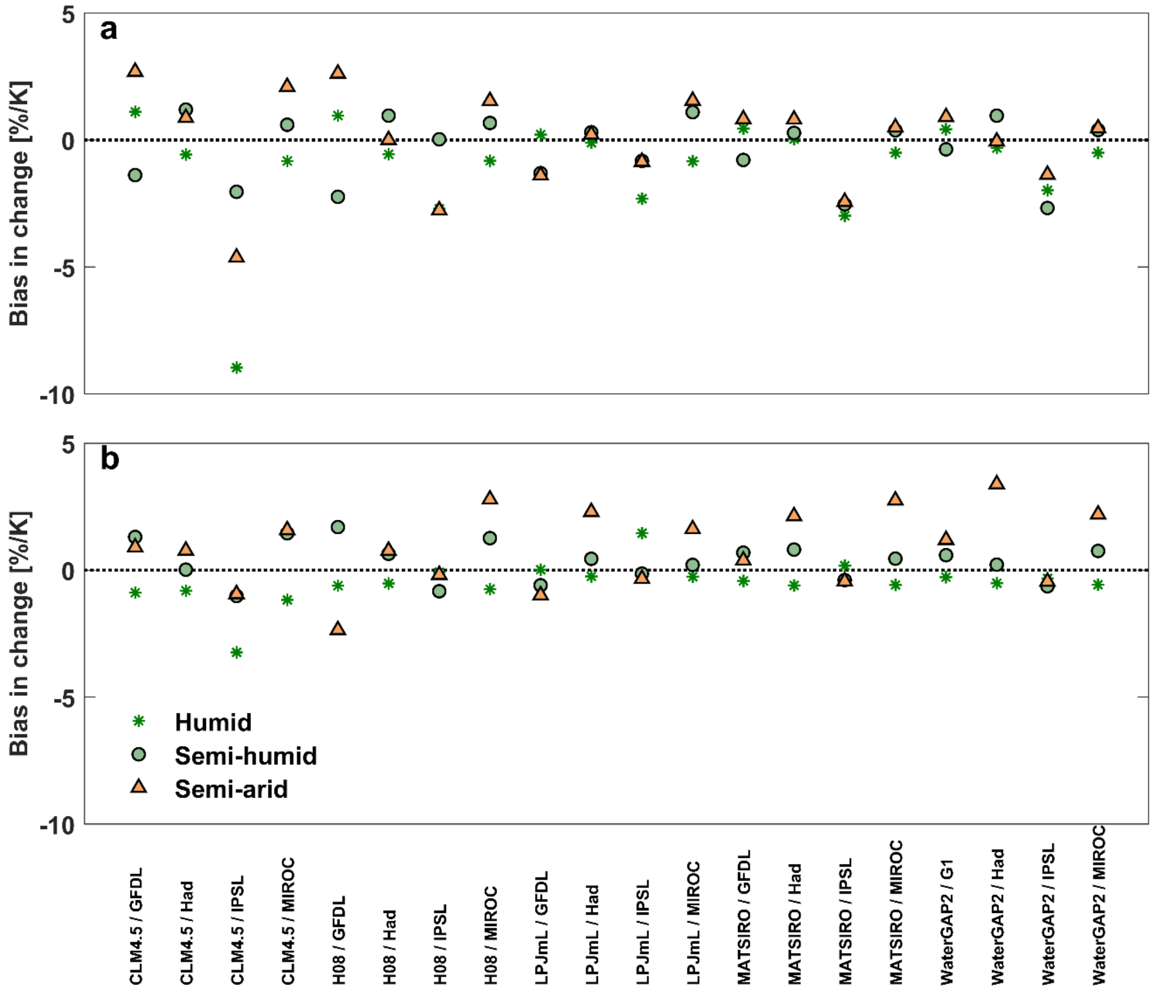

Figure S12. Bias in flood intensity changes by using (**a**) median ensemble mask and (**b**) static climate regime masks.

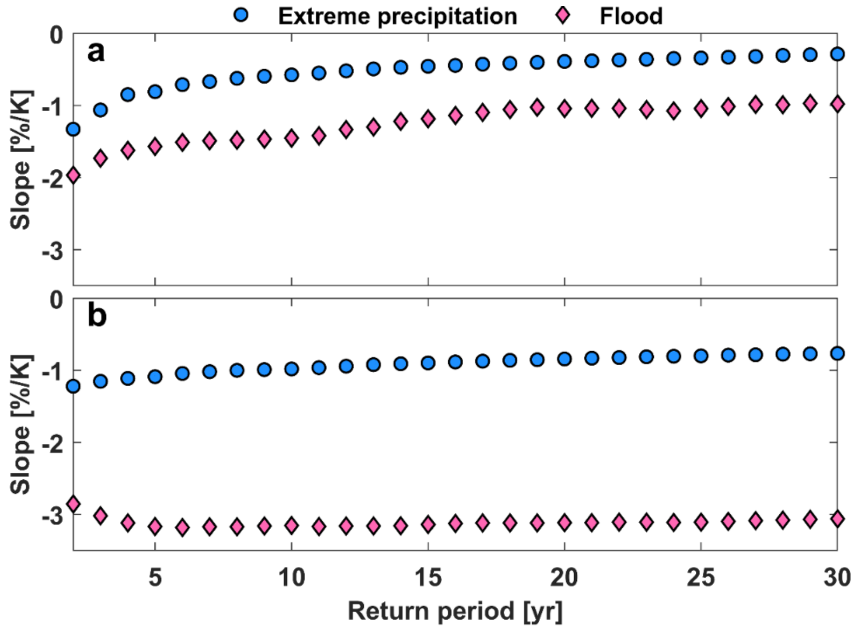

Figure S13. Regression slope of changes in extreme precipitation and flood intensity of different return periods with (**a**) climate regime and (**b**) seasons. For the slope calculations, climate regimes are arranged in the descending order of water availability as humid, semi-humid, semi-arid and arid for extreme precipitation and as humid, semi-humid and semi-arid for flood, while seasons are arranged as DJF, SON, MAM and JJA.

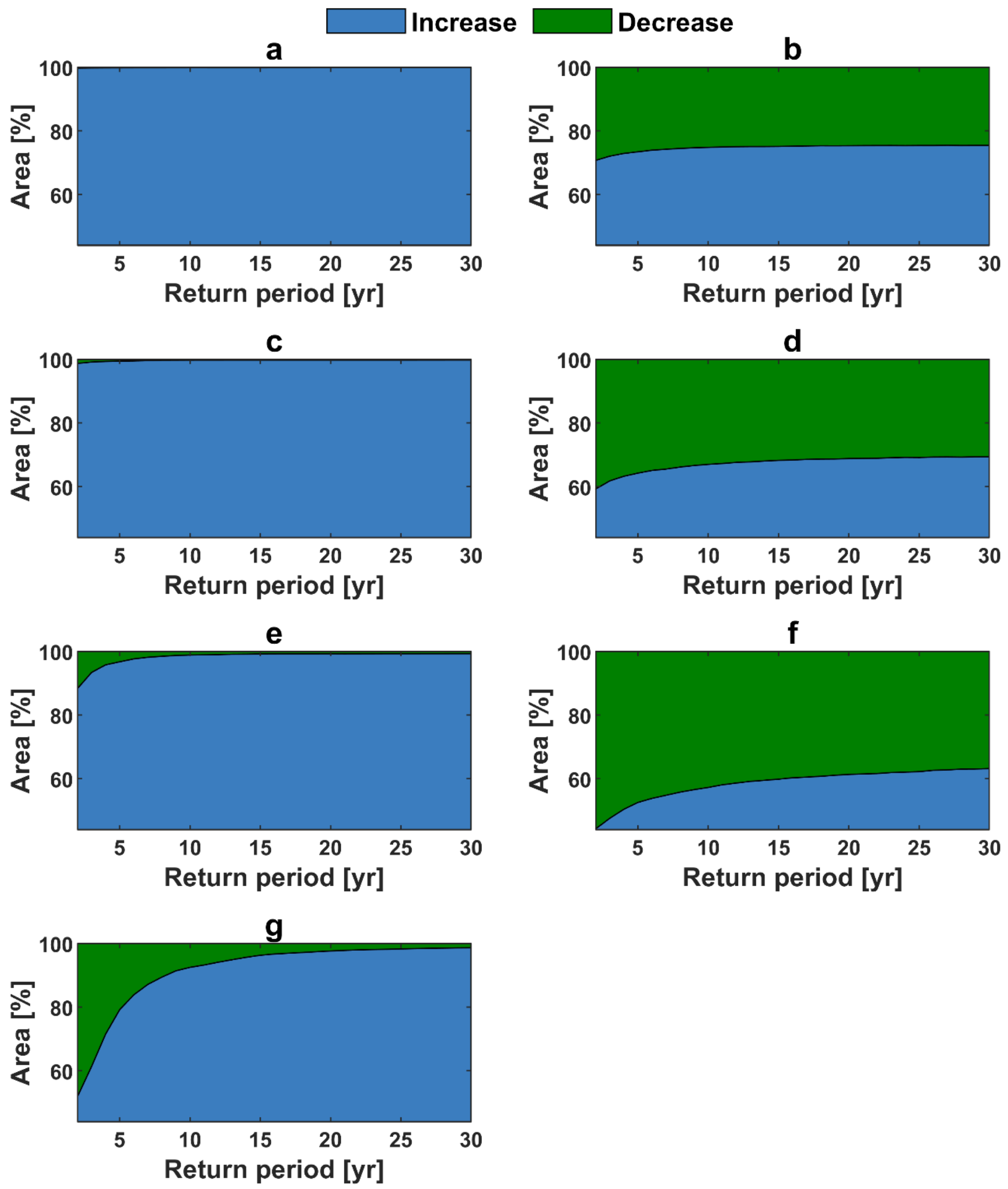

Figure S14. Percentage of (**a**, **b**) humid, (**c**, **d**) semi-humid, (**e**, **f**) semi-arid and (**g**) arid land areas with increasing and decreasing intensity signals in (**a**, **c**, **e**, **g**) extreme precipitation and (**b**, **d**, **f**) flood events with return periods ranging between 2 and 30 years based on multi-model ensemble median.

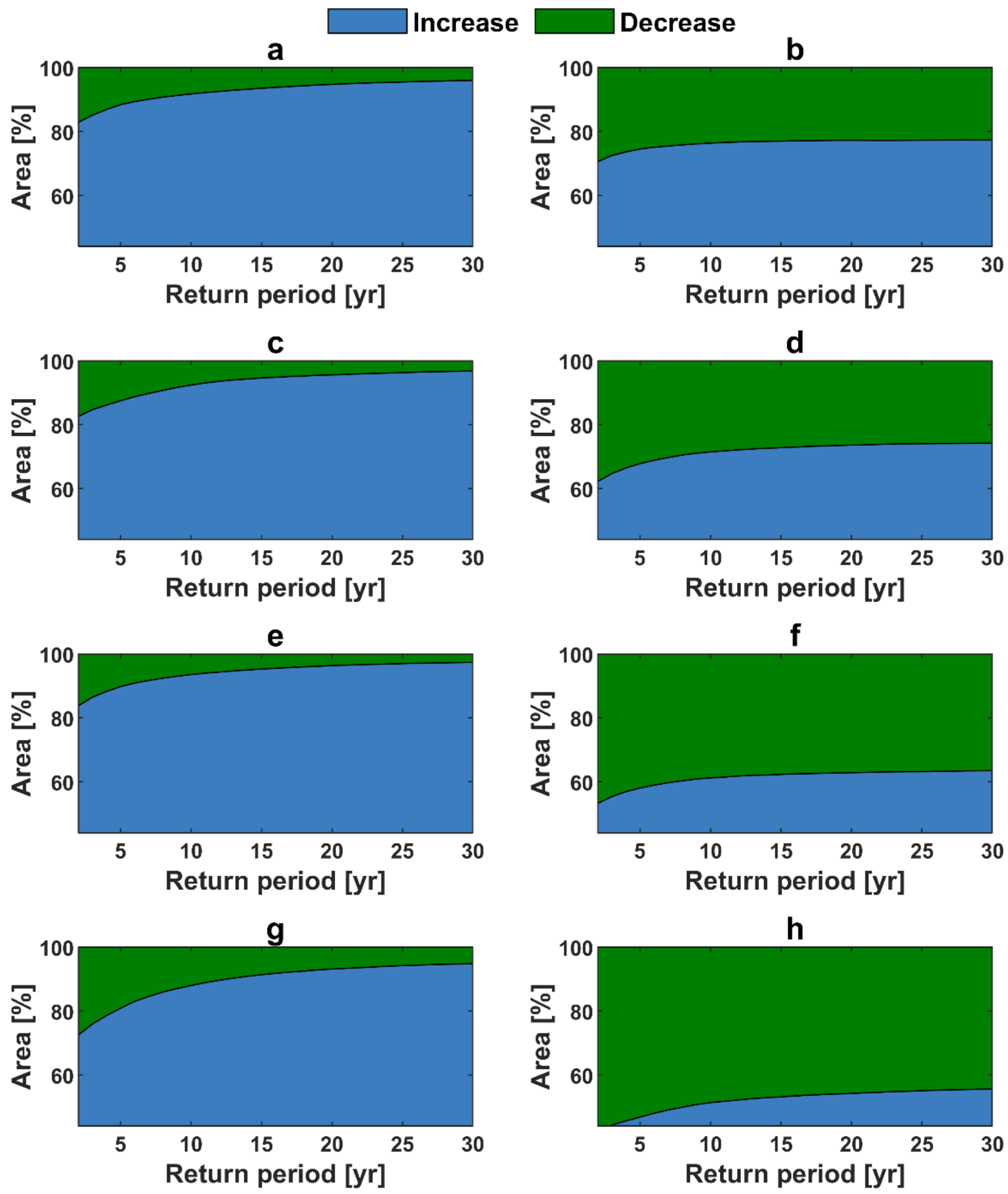

Figure S15. Percentage areas of the Northern Hemisphere mid-latitudes with increasing and decreasing intensity signals in (**a**, **c**, **e**, **g**) extreme precipitation and (**b**, **d**, **f**, **h**) flood events with return periods ranging between 2 and 30 years in (**a**, **b**) DJF, (**c**, **d**) SON, (**e**, **f**) MAM and (**g**, **h**) JJA seasons based on multi-model ensemble median.

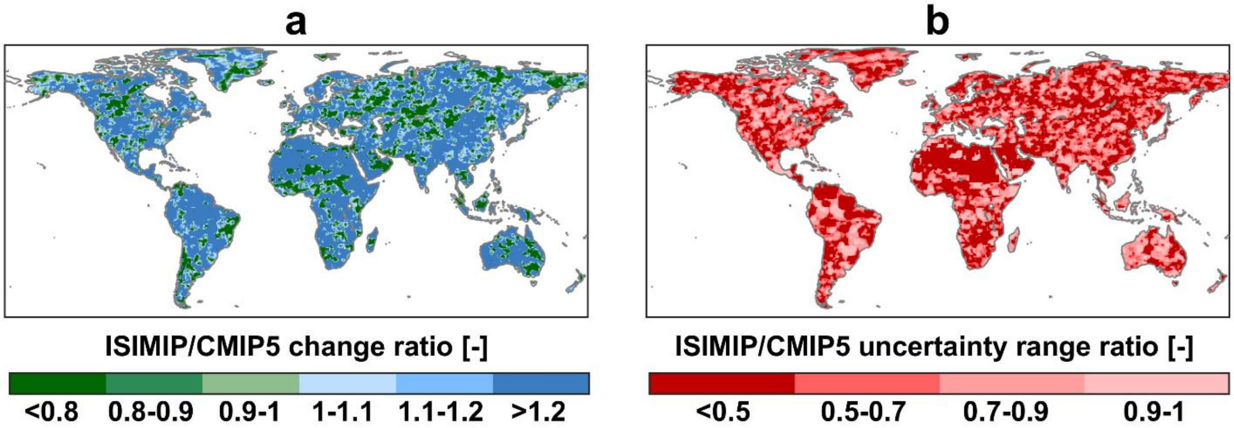

Figure S16. Comparison of (**a**) change and (**b**) uncertainty range between the four GCMs used as climate forcing in ISIMIP discharge simulations and the 24 CMIP5 GCMs used for extreme precipitation analyses. The maps were generated using the MATLAB mapping toolbox[65] (URL-https://www.mathworks.com/products/mapping.html).
